# Diversity of fungi associated with petroglyph sites in the Negev Desert, Israel, and their potential role in bioweathering

**DOI:** 10.3389/ffunb.2024.1400380

**Published:** 2024-07-05

**Authors:** Laura Rabbachin, Irit Nir, Monika Waldherr, Ylenia Vassallo, Guadalupe Piñar, Alexandra Graf, Ariel Kushmaro, Katja Sterflinger

**Affiliations:** ^1^ Institute of Natural Sciences and Technology in the Arts (INTK), Academy of Fine Arts Vienna, Vienna, Austria; ^2^ Environmental Biotechnology Laboratory, Avram and Stella Goldstein-Goren Department of Biotechnology Engineering, Ben-Gurion University of the Negev, Be’er Sheva, Israel; ^3^ Department of Bioinformatics, University of Applied Sciences, Vienna, Austria; ^4^ Laboratory of Plant Pathology, Department of Environmental Biology, Sapienza University of Rome, Rome, Italy; ^5^ School of Sustainability and Climate Change, Ben-Gurion University of the Negev, Be’er Sheva, Israel

**Keywords:** extremotolerant fungi, rock-inhabiting fungi, soil mycobiota, stone biodeterioration, ITS long-amplicon sequencing

## Abstract

The petroglyphs of the Negev Desert, Israel, are famous and valuable archaeological remains. Previous studies have investigated the microbial communities associated with petroglyphs and their potential role in stone deterioration; nevertheless, the role of fungi remains unclear. In this study, the fungal communities present on the stone and, as a comparison, in the surrounding environment (soil and air) at Negev petroglyph sites were analyzed by means of culture-dependent and -independent (metagenomic) techniques. The metagenomic results showed a high fungal biodiversity in the soil, and both approaches highlighted the prevalence of species producing melanized, large, thick-walled spores (mainly *Alternaria* spp.). From the air sampling, mostly *Cladosporium* spp. were retrieved. On the other hand, on the rock, the results seem to indicate a low presence of fungi, but with a rock-specialized mycobiota consisting of extremotolerant microcolonial fungi (MCF) (e.g., *Vermiconidia* and *Coniosporium*) and *lichens* (*Flavoplaca*). In addition, low proportions of cosmopolitan fungi were detected on the stone, but the comparison of the data clearly indicates that they are transients from the surrounding environment. The ability of the isolated strains to dissolve CaCO_3_ and therefore be a potential threat to the petroglyphs (limestone substrate) was tested, but only one strain resulted in positive acid production under laboratory conditions. Nevertheless, both lichens and MCF detected in this study are well-known stone deteriogens, which may have a significant impact on the petroglyph’s deterioration.

## 1 Introduction

**Figure 1:**
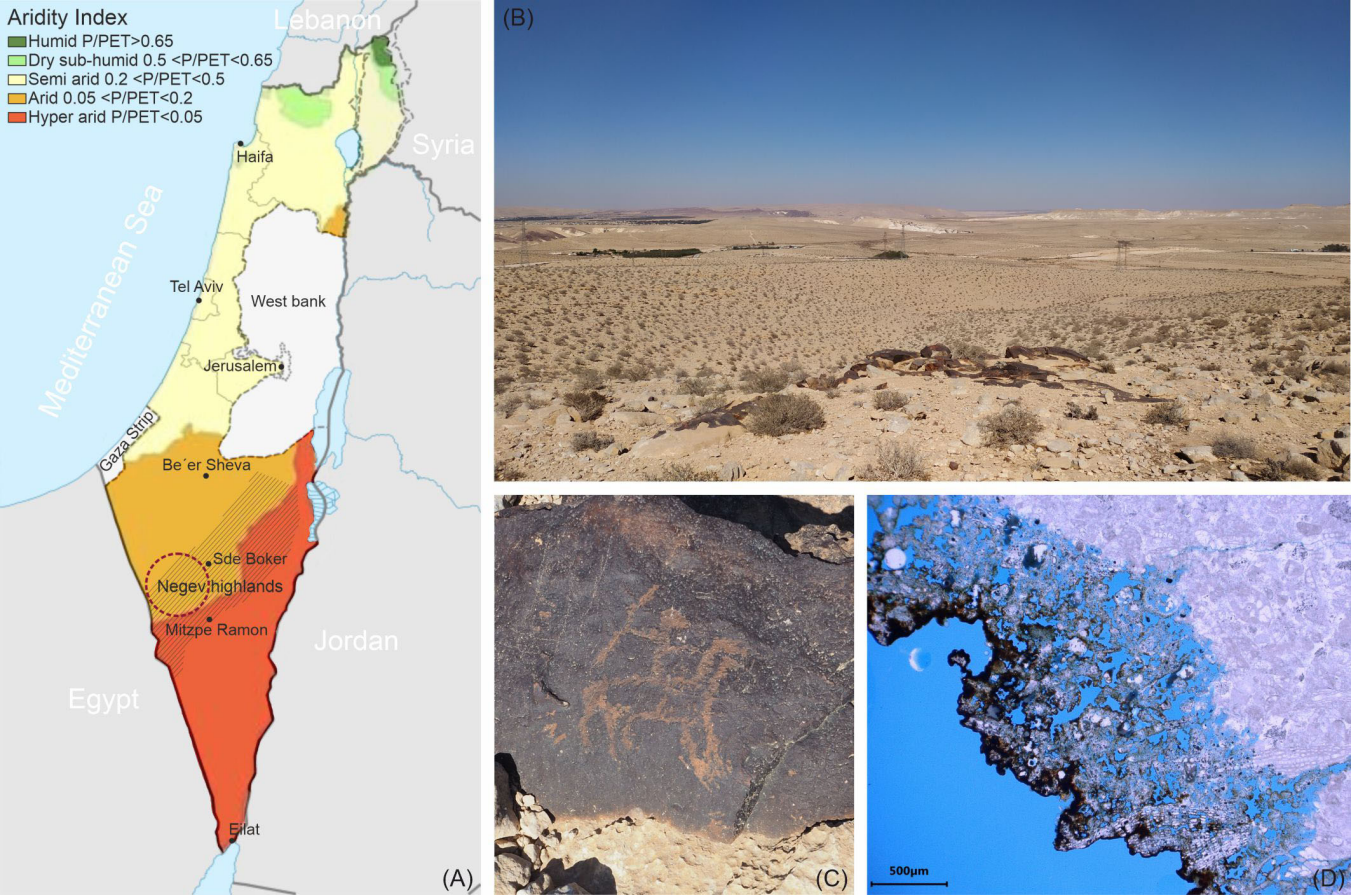
**(A)** Map of Israel showing different aridity index (AI) zones (AI is calculated as average annual precipitation (P)/potential evapotranspiration (PET)) (adapted from Chen, 2017) and the location of the petroglyph sites considered for this study (maroon circle) in the Negev Highlands. **(B)** A view of the Negev Desert with a petroglyph site, and **(C)** an example of a petroglyph carved into the desert varnish coating the stone. **(D)** A petrographic thin section of a stone sample from the petroglyph sites showing the weathered bedrock beneath the black coating.

The Negev Desert of Israel is home to thousands of petroglyph sites, dating back at least 5,000 years (Eisenberg-Degen and Nash, 2014), representing a precious remnant of past civilizations that inhabited the desert long ago (Eisenberg-Degen et al., 2022). The petroglyphs are mostly located in the Negev Highlands (**Figures 1A, B**) and are carved into a natural thin, dark coating known as desert varnish (Perry et al., 2006; Andreae et al., 2020), covering some of the local limestone formations (**Figure 1C**). Despite the harsh and inhospitable conditions, deserts around the world are known to harbor a multitude of extremotolerant and extremophilic microorganisms, including fungi that were once thought to be less resilient than prokaryotes (Gostinčar et al., 2023). These microorganisms have adapted to withstand extreme stress factors such as very high temperatures, temperature fluctuations, high ultraviolet and infrared irradiation, low organic matter content, and extremely limited availability of water (Sterflinger et al., 2012). Consequently, even the petroglyphs of the Negev Desert undergo biogenic weathering, like any other natural rock surfaces or stone cultural heritage exposed to the environment (e.g., Steiger et al., 2014; Favero-Longo and Viles, 2020). Recent studies have investigated the biodiversity of lithobiontic microbial communities associated with petroglyph sites in the Negev Desert and their potential role in the deterioration of this valuable cultural heritage (Nir et al., 2019, Nir et al., 2021; Rabbachin et al., 2022; Nir et al., 2023). These investigations showed deterioration patterns of the rock substrate, revealing a clear weathered layer underneath the dark coating (**Figure 1D**) due to the complete lack of the stone calcite matrix in these areas (Nir et al., 2021; Rabbachin et al., 2022), indicating a feasible dissolution of the calcareous material by microbial activity (Krumbein and Jens, 1981; de Los Ríos et al., 2009). Metagenomic analyses of the stone microbial community, although bacteria-dominated, also included fungi (Nir et al., 2021; Rabbachin et al., 2022). However, most of the genera identified did not appear to be rock inhabiting species (except for a few taxa such as *Exophiala*, *Coniosporium*, and *Letharia*) but rather cosmopolitan airborne fungi, with minimal evidence of fungi growing on stone found through microscopic observations (Rabbachin et al., 2022). Various studies have reported airborne ubiquitous species as part of the desert endolithic communities (Gonçalves et al., 2016; Tang et al., 2016; Gómez-Silva et al., 2019; Grishkan and Temina, 2023), but this could be the result of “post-sampling contamination” (Coleine et al., 2021) or the presence of airborne spores deposited on the rock surface (Sterflinger et al., 2012). In fact, among the variety of extremotolerant and extremophilic microorganisms that inhabit the desert, fungi are specialized to resist complete desiccation through the formation of thick-walled spores (Sterflinger et al., 2012), allowing them to survive in a dormant state even in the driest environments (e.g., the Atacama Desert, Chile) (Conley et al., 2006). In the last decade, with the development of novel molecular techniques, studies on the soil mycobiota of deserts worldwide have revealed unexpectedly rich biodiversity (Zhang et al., 2016; Murgia et al., 2018; Santiago et al., 2018; Ameen et al., 2021). Griskhan and colleagues have conducted extensive research on the soil microfungi of Israel’s Negev Desert (Grishkan, 2019; Grishkan et al., 2023), highlighting that fungi with thick-walled, strongly melanized spores (e.g., *Cladosporium* and *Chaetomium*) and large multicellular conidia (e.g., *Alternaria*) were dominant in the majority of the Negev topsoil, while aspergilli were a fundamental part of the thermotolerant fungal community.

Compared to the soil mycobiota, desert lithobionts have received less attention, and a comprehensive understanding of both their diversity and ecological function is still in progress (Coleine et al., 2022). It is within this category, however, that the real specialists of the desert environment are found, not only able to survive but also to thrive in the lithic support (Sterflinger et al., 2012). Together with lichens, these rock specialists are the so-called microcolonial fungi (MCF) or black fungi, also referred to as rock-inhabiting fungi (RIF), when they colonize stone substrates (Liu B. et al., 2022 and references therein). This peculiar group of microorganisms is extremely resilient to hostile environments, and species belonging to this group have been isolated from hot deserts (Staley et al., 1989) to extremely cold Antarctic drylands (Selbmann et al., 2015; Coleine et al., 2021), but also in more moderate climates like the Mediterranean basin, where they have often been associated with the deterioration of stone cultural heritage (Sterflinger and Krumbein, 1997; Marvasi et al., 2012; Onofri et al., 2014; Isola et al., 2016; Paiva et al., 2023). In fact, MCF, due to their great ability to penetrate and degrade hard rocks as well as softer stones like limestone, sandstone, and marble (often used as building construction material or artistic support), is considered highly dangerous for stone artifacts. They can cause black discoloration, surface exfoliation (sugaring) due to their inter-crystalline growth, and surface erosion (biopitting) that can eventually lead to large cavities (De Leo et al., 2022). As well as MCF, lichens are also well-known to be rock deteriogens and a potential threat to stone cultural heritage (e.g., de Los Ríos et al., 2009; Cozzolino et al., 2022).

In this study, we aimed to better understand whether fungi associated with petroglyphs in the Negev Desert are transient or integral members of the microbial ecosystem. We analyzed the biodiversity of fungal communities on the rock and compared it to those in the surrounding environment (soil and air). To achieve this, we utilized high-throughput sequencing technology (nanopore sequencing) targeting the Internal transcribed spacer (ITS) fungal region alongside culture-based methods. In addition, to gain deeper insights into the potential role of fungi in the deterioration of petroglyphs (carbonate rock), the ability of the isolated strains to dissolve calcium carbonate was tested.

## 2 Materials and methods

### 2.1 Study location and sampling

Samples were collected from petroglyph sites in the central-west Highlands of the Negev Desert, located in the southern part of Israel (**Figure 1A**). The Negev Highlands are classified as an arid (0.05<P/PET<0.2) to hyperarid (P/PET<0.05) climatic zone (**Figure 1A**), with a yearly average rainfall amount of 87 mm, mostly during the winter season. The annual average temperature is 19°C; the average daily maximum during the hottest months is 32°C, and the average daily minimum during the coldest months is 16.5°C, whereas the average of the daily minimums is 5°C in the coldest and 18°C in the hottest months. The absolute minimum registered in winter was -4.5°C, while the absolute maximum in summer was above 43°C. Meteorological data were gathered from the Israel Meteorological Service (IMS), Sde Boker station (30°52′25″ N, 34°47′35″ E) (Israel Meteorological service, 2024). Previous climatic measurements conducted with an *in situ* monitoring system equipped with thermocouples showed that the rock temperature in summer can reach up to 56.3°C and be considerably higher than the average air temperature (Rabbachin et al., 2022).

For a complete examination of the fungal communities present in both the stone and the surrounding environment at the petroglyph sites, samples were taken from limestone rocks coated by desert varnish, adjacent to the petroglyphs, and from the soil in proximity of the rock sampling points. One sample was collected from a stone on which the black coating was absent, and only an orange layer, usually present between the white limestone bedrock and the black varnish (see Nir et al., 2021; Rabbachin et al., 2022), was visible. The rock samples were labeled AV_A, AV_B, EZ_A (black coating), and EZ_D (orange layer), while the corresponding soil samples were labeled AVS_A, AVS_B, EZS_A, and EZS_D. In addition, five sampling points were chosen for air sampling using the sedimentation method, which is based on the use of Petri dishes being exposed to air for a defined period of time (Viani et al., 2020). Malt extract agar (MEA) (2% malt extract, 0.1% peptone from casein, 2% glucose anhydrous, and 1.5% agar) and dichloran-glycerol (DG18) agar base (3% GranuCult™ Dichloran-Glycerol chloramphenicol agar base (Merck, Germany), 22% glycerol) plates were left open on the ground for 4 h. Upon completion, the air sampling plates were transferred to the laboratory at Ben-Gurion University of the Negev (Israel). Soil and stone samples were placed in sterile plastic bags or tubes and transferred to the microbiology laboratory at the Academy of Fine Arts of Vienna (Austria) for molecular analysis and direct fungal isolation through cultivation methods.

### 2.2 Cultivation strategy and identification of fungal isolates

#### 2.2.1 Isolation of culturable fungi

For soil and stone samples, 1 g of each sample (rock samples were previously crushed with a sterilized mortar and pestle) was suspended by vortexing in 5 mL of sterile 0.9% (w/v) NaCl solution with Tween 80 (0.001%) and homogenized for 1 h on the shaker (Thermo Fisher Scientific, MaxQ™ 2000). From the suspension, serial dilutions were prepared, and 100 µL of the solutions were plated in triplicates on MEA and DG18 plates (recipes described above in Section 2.1). The plates were incubated at room temperature (range: 22°C–24°C) for 10 days and up to 20 days for slow-growing fungi. The colony-forming units (CFUs) were calculated from the mean value of the three replicas on MEA and DG18 per gram of sample. Individual colonies were selected based on their color and morphology and transferred to fresh plates containing the same medium to obtain pure isolates. The isolates were observed macroscopically and microscopically (Olympus BX51 light microscope, Olympus Optical Co., Ltd., Tokyo, Japan) and divided into categories by appearance and morphology. Isolates that exhibited identical morphological characteristics were grouped (20 groups in total), and, per group, one isolate was selected and temporarily preserved on agar slants at 4°C for further molecular identification by Sanger sequencing.

Petri dishes used for air sampling were incubated at room temperature (range: 22°C–24°C) for 1 week. After the incubation period, colonies were counted, observed under the microscope, and transferred to fresh plates to obtain pure isolates.

#### 2.2.2 DNA extraction, sequencing, and taxonomic analysis of isolated strains

The total DNA from the fungal isolates found in the air was extracted using the DNeasy PowerSoil Pro Kit (Quiagen, Hilden, Germany). Total DNA from selected isolates from stone and soil was instead extracted using the DNeasy Plant Pro Kit (Qiagen, Hilden, Germany) according to the manufacturer’s instructions with the following adaptation: The lysing step was carried out with the FastPrep-24 bead beater (MP Biomedicals, USA) with three cycles of 60 s at a speed of 5 m/s. The DNA yields were quantified using a Qubit 2.0 fluorometer (Thermo Fisher Scientific, Waltham, MA, USA) with the Qubit dsDNA HS Assay Kit. The fungal ITS region, corresponding to the ITS1 and ITS2 regions along the 5.8S rRNA gene, was amplified using the universal primers ITS1 forward and ITS4 reverse (White et al., 1990). The Promega (Madison, Wisconsin, USA) premixed PCR Mastermix, 2X [50 units/mL of Taq DNA polymerase supplied in a proprietary reaction buffer (pH 8.5), 400-µM dATP, 400-µM dGTP, 400-µM dCTP, 400-µM dTTP, and 3-mM MgCl_2_] was diluted to 1X and 0.5 pmol/µL of each primer (stock: 50 pmol/μL) was added. In a total volume of 50 µL, between 10 ng and 30 ng of template DNA was added. PCR was performed in a BioRad C1000 Thermal Cycler with the following program: 3 min at 95°C, followed by 35 cycles of 1 min at 95°C, 30 s at 55°C, and 1 min at 72°C, with a final extension step of 5 min at 72°C. The resulting PCR products were purified using the QIAquick PCR Purification Kit (Qiagen, Hilden, Germany) and analyzed by electrophoresis on a 2% (w/v) agarose gel. Sanger sequencing of the PCR products was outsourced to Eurofins Genomics (Vienna, Austria) (for stone and soil isolates) and at the BGU Genomics unit (for air isolates) using primers ITS1F and ITS4R. The resulting nucleotide sequences were compared to those in the online databases provided by the National Center for Biotechnology Information (NCBI) using the BLAST search tool. The newly generated ITS sequences from all isolated strains have been deposited in the NCBI database under the accession numbers PP464157–PP464193 (**Table 1**).

**Table 1:** Identification of the fungal strains isolated from rock, soil, and air from the Negev Desert petroglyph sites.

Strain	Closest identified organism (NCBI GenBank accession number^a^)	Similarity (%)	Isolation source	NCBI accession number^b^
*NS1*	*Alternaria multiformis* isolate 177_1_ITS1F (OR781415.1)*/Alternaria consortialis* isolate UMBKKU1 (OQ360812.1) Alternaria sect. Ulocladioides	99.8	Stone and soil	PP464157
*NS2*	*Alternaria terricola* strain GRSH53 (KY788070.1)/*Alternaria obovoidea* strain ICMP 1145 (OR543735.1) Alternaria sect. Ulocladioides	100/99.8	Soil	PP464158
*NS3*	*Alternaria obovoidea* strain HSAUP_XF031144 (AY762942.1)/*Alternaria atra* strain ICMP 1144 (OR543716.1) Alternaria sect. Ulocladioides	100/99.8	Soil	PP464159
*NS4*	*Alternaria alternariae* culture CBS:126989 strain CBS 126989 (MH864321.1)/*Alternaria botrytis* genomic DNA (OW982330.1) Alternaria sect. Ulocladium	100	Soil	PP464160
*NS5*	*Alternaria chlamydosporigena* isolate *CK391* (MH474263.1)*/Alternaria tellustris* voucher culture Y211B (MW791841.1) Alternaria sect. Embellisia	100	Soil	PP464161
*NS6*	*Alternaria chlamydosporigena* isolate *CK391* (MH474263.1)/*Alternaria tellustris* voucher culture Y211B (MW791841.1) Alternaria sect. Embellisia	100	Soil	PP464162
*NS7*	*Alternaria cumini* strain CF-090366 (MG065793.1) Alternaria sect. Eureka	99.6	Soil	PP464163
*NS8*	*Alternaria tenuissima* isolate DD-ZX-TSC3 (OQ026924.1)/*Alternaria alternate* isolate DD-DG-HL1 (OQ026859.1) Alternaria sect. Alternaria	100	Soil	PP464164
*NS9*	*Alternaria phragmospora* (JQ796758.1)*/Alternaria mouchaccae* genomic DNA (OW983428.1) Alternaria sect. Phragmosporae	99.3	Soil	PP464165
*NS10*	*Alternaria alternata* isolate JM19 (PP094555.1)/*Alternaria tenuissima* Alternaria sect. Alternaria	99.6	Soil	PP464166
*NS11*	*Alternaria malorum* strain BLE14 (FN868458.1) Alternaria sect. Chalastospora	99.1	Soil	PP464167
*NS12*	*Alternaria* sp. isolate NB661 (OK353793.1)/*Alternaria hungarica* strain AUMC 14101 (MN883928.1)	100/99.8	Soil	PP464168
*NS13*	*Alternaria* sp. isolate NB661 (OK353793.1)/*Alternaria hungarica* strain AUMC 14101 (MN883928.1)	100/99.8	Soil	PP464169
*NS14*	*Coniosporium* sp. isolate MA 4614 (AJ972790.1)	99.2	Stone	PP464170
*NS15*	*Dimorphosporicola* sp. strain CF-090768 (MG065837.1)	98.9	Soil	PP464171
*NS16*	*Preussia australis* strain AV1S14–2 (KP101202.1)	100	Soil	PP464172
*NS17*	*Botryotrichum piluliferum* sample 15 (LC745679.1)	99.8	Soil	PP464173
*NS18*	*Xenodidymella* sp. strain A SK-2018 strain T324I1 (MK100160.1)	99.8	Soil	PP464174
*NS19*	*Cladosporium halotolerans* strain AUMC 11387 (MN826823.1)/*Cladosporium sphaerospermum* genes (AB572902.1)	99.8/99.5	Soil	PP464175
*NS20*	*Cladosporium cladosporioides* strain JA14 (KF417590.1)/*Cladosporium allicinum* culture CPC:22349 (MF472904.1)	99.8	Stone and soil	PP464176
*NA1*	*C. cladosporioides* complex	100	Air	PP464177
*NA2*	*C. cladosporioides* complex	100	Air	PP464178
*NA3*	*Cladosporium cladosporioides* strain WA0000019047 (JX981454.1)/*Cladosporium uwebraunianum* genomic DNA (OW987346.1)	99.8	Air	PP464179
*NA4*	*C. herbarum* complex	100	Air	PP464180
*NA5*	*Cladosporium limoniforme* culture DTO:305-G4 (MF473139.1) *Cladosporium tenellum* strain 2.5.46 (KX674650)	100	Air	PP464181
*NA6*	*Stemphylium vesicarium* genomic DNA (OW984035.1)	99.8	Air	PP464182
*NA7*	*Penicillium glandicola* strain F-91 (MF077229.1)	99.7	Air	PP464183
*NA8*	*Nigrospora osmanthi* isolate BC301.13 (OQ518439.1)/*Nigrospora sphaerica* strain DYB5 (MK482388.1)	100/99.8	Air	PP464184
*NA9*	*Nigrospora osmanthi* isolate BC301.13 (OQ518439.1)*/Nigrospora sphaerica* strain DYB5 (MK482388.1)	100/99.8	Air	PP464185
*NA10*	*Botrytis fabae* strain DH-7 (MN589852.1)/*Botrytis cinerea* strain SJH-2 (MN589849.1)	100	Air	PP464186
*NA11*	*Fusarium equiseti* isolate RM6 (MG664737.1)	99.8	Air	PP464187
*NA12*	*Cladosporium sphaerospermum* genomic DNA (LN834388.1)/*Cladosporium lignicola* strain ATCC (AF393709.2)	100	Air	PP464188
*NA13*	*Cladosporium sphaerospermum* strain WL5–1A (MF422149.1)	100	Air	PP464189
*NA14*	*Cladosporium xanthochromaticum* genomic DNA (OW983633.1*)/Cladosporium perangustum* isolate ES-31–3 (MZ568189.1)	100/99.8	Air	PP464190
*NA15*	*C. herbarum* complex	99.3	Air	PP464191
*NA16*	*Cladosporium cladosporioides* strain MCCC3A00182 (MT258647.1)/*Cladosporium xanthochromaticum* isolate 32 (MK311276.1)	100	Air	PP464192
*NA17*	*Alternaria* sp. HT-M18-L (KJ527009.1)/*Alternaria brassicicola* isolate Ab9H (KF542557.1)	99.6	Air	PP464193

^a^Accession number of the closest identified organism in the NCBI GenBank database; ^b^Accession number of the strains isolated in this study and deposited in the NCBI GenBank database.

### 2.3 Biodegradative plate assay: calcium carbonate dissolution

The potential of fungal strains isolated from soil and rock to solubilize calcium carbonate was tested on CaCO_3_ glucose agar medium (CaCO_3_ 0.5%, glucose 1%, and agar 1.5%; pH adjusted to 8.0 with 1 M HCl) (Albertano and Urzí, 1999; Pangallo et al., 2012). The plates were inoculated in triplicate with fungal mycelium and incubated at room temperature (range: 22°C–24°C) for 3 months. The positive strains displayed a clear zone around the colonies, confirming the dissolution of CaCO_3_.

### 2.4 Metagenomic analysis

#### 2.4.1 DNA extraction, pre-amplification of the fungal ITS region, library preparation, and nanopore sequencing

For the culture-independent study, total DNA was extracted directly from four stone samples (AV_A, AV_B, EZ_A, and EZ_D) and from four soil samples (AVS_A, AVS_B, EZS_A, and EZS_D) using the FastDNA Spin Kit for soil (MP Biomedicals, Illkrich, France) according to the recommendations by the manufacturer. For each sample, two DNA extractions with 0.5 g of crushed stone or soil were performed, and the obtained DNA was pooled and quantified using a Qubit 2.0 fluorometer (Thermo Fisher Scientific, Waltham, MA, USA) with the Qubit dsDNA HS Assay Kit. The fungal ITS region was pre-amplified as described in Section 2.2.2, and the obtained PCR products were purified using the QIAquick PCR Purification Kit. Library preparation and sequencing of the ITS long-amplicons were performed according to the Oxford nanopore four-primer PCR protocol in combination with the PCR Barcoding Kit (SQK-PBK004), as described by (Tichy et al., 2023), but customized for fungi. This involved using ITS1F and ITS4R as native primers, an annealing temperature of 55°C, and an extension temperature of 65°C for 45 s. The barcoded samples were pooled in the desired ratios to achieve a total molar concentration ranging between 50 fmol and 100 fmol in a 10-µL volume. The prepared DNA library was loaded into the flow cell (SpotOn Flow Cell Mk I R9 Version, FLO‐MIN 106D), and sequencing was performed for 48 h in the MinIon Mk1C device.

#### 2.4.2 Sequence analysis

Basecalling of the fast5 files was conducted with the Guppy Basecalling Software (Oxford Nanopore Technologies, Limited) Version 5.0.11 + 2b6dbff with the dna_r9.4.1_450bps_hac model. From the acquired reads, the remaining adapters were removed, and chimeric sequences were split using porechop (version 0.2.4). Reads were then filtered with NanoFilt (version 2.8.0) to eliminate low-quality read ends (40 bases trimmed at both 5’ and 3’ ends) and to obtain quality scores (QS) > 9 as well as read lengths between 300 and 900 bases (the expected amplicon length). After filtering, the median QS ranged from 11.4 to 14.3, representing error rates between 7.24% and 3.72%. Metataxonomic classifications were performed with Emu (version 3.4.4) using the provided pre-built UNITE general fasta v8.3 fungi database, which contains the RepS/RefS of all SHs (created on 10 May 2021). Visualization of the classification results was carried out in R (4.3.1) using the packages pheatmap, tidyverse, and RColorBrewer. Relative abundance cutoffs were set at 0.1% or 0.5% on the taxonomic level displayed, with all classifications below that threshold labeled as “others.” All data are accessible on the NCBI BioProject PRJNA1078577.

## 3 Results

### 3.1 Cultivation analyses and taxonomic identification

After an incubation period of up to 20 days, the viable titer of fungi in the soil samples ranged from 3.3 CFU/g × 103 CFU/g to 1.5 CFU/g × 104 CFU/g on MEA and from 2.1 CFU/g × 103 CFU/g to 8.3 CFU/g × 103 CFU/g on DG18. Fungi could only be cultivated from two rock samples on MEA (AV_B and EZ_D), with a viable titer of 1.5 CFU/g × 103 CFU/g and 1.2 CFU/g × 103 CFU/g, and from one sample (EZ_D) on DG18 (1.0 CFU/g × 103 CFU/g). The number of colonies counted on the air sampling plates varied from 56 to 115 on MEA and from 61 to 92 on DG18.

A total of 58 isolates were retrieved from the soil samples (IS1-IS58, **Supplementary Table 1**), 6 isolates were obtained from the rock samples (IR1-IR6, **Supplementary Table 1**), and 17 isolates (NA1-NA17) were obtained from the air sampling. As explained in Section 2.2.1, since some of the isolates were found to be identical in morphology and appearance, only 20 strains (labeled as NS1–NS20) were selected to be sequenced from the stone and soil samples, while all 17 strains (labeled as NA1–NA17) were sequenced from the air sampling. Pictures of some of the isolated strains are reported in **Supplementary Figure 1**. In addition, some strains from the soil, such as Epicoccum spp., Penicillium spp., Aspergillus spp., and Aurobasidium pollulans, and one strain of MCF from stone were solely identified through microscopic examination and not subjected to sequencing. A list of all isolates from rock and soil showing the division into groups is reported in **Supplementary Table 1**, while the results of the taxonomic identification are summarized in **Table 1**.

The 37 strains sequenced were affiliated with 12 different genera, all belonging to the phylum Ascomycota. From the soil samples, the genus Alternaria was dominant and had the highest number of species (**Table 1**), while the genus Cladosporium was also frequently occurring but presented lower biodiversity. The genera that occurred less frequently in soil and were represented only by one species were Preussia and Botryotrichum, together with the genera Dimorphosporicola and Xenodidymella, which occurred rarely. From the rock samples, three different genera were retrieved: Alternaria and Cladosporium (strains NS1 and NS20), also occurring in the soil, and Coniosporium, which was instead only retrieved from the stone. Concerning air sampling, the genus Cladosporium was clearly dominant, with almost 60% of the strains identified belonging to this genus. In addition, species of the genera *Stemphylium, Penicillium, Nigrospora, Botrytis, Fusarium,* and *Alternaria* were also identified.

The species assignment for strains of *Alternaria* and *Cladosporium* was often ambiguous due to the low variety of the ITS region of these genera. Therefore, in cases of ambiguous identification, the *Alternaria* section to which the strain belongs was added (**Table 1**), according to the NCBI taxonomic classification. *Cladosporium* strains were assigned to a *Cladosporium* complex, according to Bensch et al (Bensch et al., 2012), and no accession number of a single strain as the closest identified organism is given (**Table 1**).

### 3.2 Dissolution of calcium carbonate

To investigate the potential role of fungi in the deterioration of limestone, the ability to solubilize CaCO_3_ of the identified fungal strains isolated from stone and soil samples from Negev petroglyph sites was tested on CaCO_3_ glucose agar medium. Only the strain NS16, identified as *Preussia australis*, tested positive, showing a clear halo around the colony indicating the dissolution of calcium carbonate (**Supplementary Figure 2**).

### 3.3 Metagenomic analysis

For a comprehensive characterization of the mycobiome associated with the stone harboring petroglyphs and the surrounding environment, targeted ITS long-amplicon sequencing with nanopore technology was conducted. The DNA yield extracted from the rock samples ranged from 7 ng/µL to 14.5 ng/µL, while higher yields between 32 ng/µL and 85 ng/µL were obtained from the soil samples. The eight samples were barcoded and used to prepare DNA libraries, which were subsequently loaded and sequenced in a single flow cell. The total reads generated per barcoded sample ranged from 205,400 to 407,400, except for one sample (EZ_A) that registered only 23,718 reads. After processing the raw reads (as described in Section 2.4.2), three samples (AV_A, AV_B, and EZ_A, all from rock coated with black varnish) did not pass quality control since almost no sequences with the correct length (300–900 bp) were present. Therefore, taxonomical classification was performed only on the five samples (AVS_A, AVS_B, EZS_A, EZS_D, and EZ_D), where ITS sequences were successfully amplified by PCR analysis (see **Supplementary Table 2**). For these samples, the number of filtered reads ranged from 122,005 to 210,224, with an average fragment length of 496. Details about the sequencing data are available in **Supplementary Table 2**.

**Figure 2:**
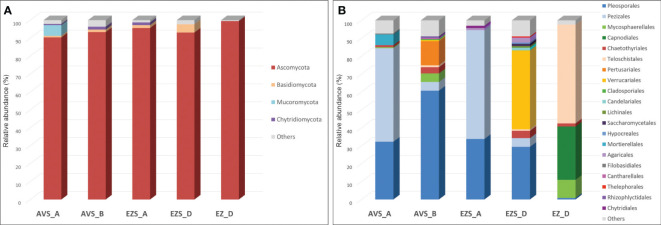
Stacked column charts showing the relative abundance of the fungal communities in each sample **(A)** at the phylum level (cutoff 0.5%) and **(B)** at the order level (cutoff 0.5%). “Others” refers to all classifications that have either been detected under the 0.5% threshold or have not been identified at that taxonomic level but only at higher ones.

The analysis of the DNA sequencing data that could be phylogenetically assigned revealed that the phylum Ascomycota was dominant, representing between 90% and 96% of the total classified reads in the soil samples and 99.5% in the rock sample (EZ_D), with no other phyla identified above 0.5%. The phylum Basidiomycota was present in all soil samples, ranging from 1% to 5%, while the phylum Chytridiomycota accounted for less than 2% (samples AVS_A, AVS_B, and EZS_D). The phylum Mucoromycota was only present in sample AVS_A, with a relative abundance of 6% (**Figure 2A**).

**Figure 3:**
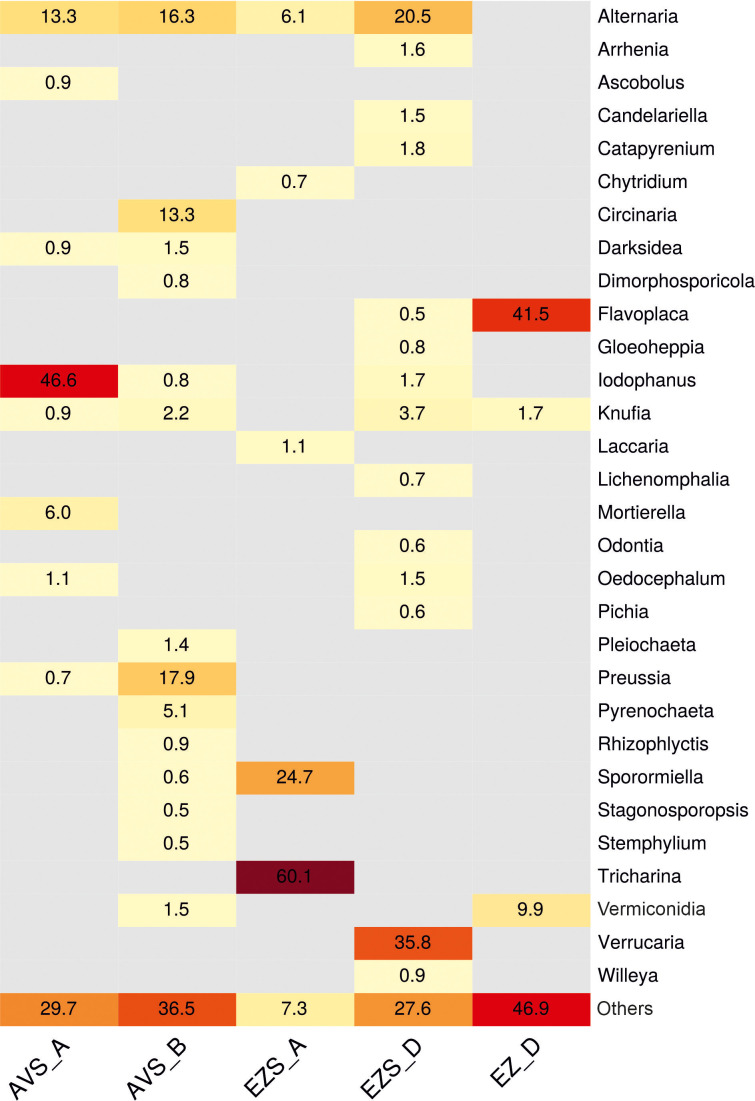
Heatmap displaying the relative abundance (%) of the fungal communities at the genus level in each sample (0.5% cutoff). “Others” refers to all classifications that have either been detected under the 0.5% threshold or have not been identified at the genus level but only at a higher one.

The relative abundance of the fungal communities at the order level (0.5% cutoff) is reported in **Figure 2B**. Due to the detection of many fungal genera in low abundance (< 0.5%), a heatmap of the genera with a cutoff of 0.5% is shown in **Figure 3** for a better visualization of the results. The heatmap with a cutoff of 0.1% is reported in the **Supplementary Material** (**Supplementary Figure 3**), along with a heatmap of the fungal species detected in each sample (**Supplementary Figure 4**).

Within the phylum Ascomycota, the order Pleosporales was present in all samples (**Figure 2B**), with a relatively high abundance ranging from 29% to 61% in the soil samples and a low abundance in the rock sample (0.8%). Within this order, the genus Alternaria was consistently detected in all soil samples (**Figure 3**) with variable proportions (6%–20.5%) and was also present in the stone sample, but below 0.5%. The genus Sporormiella was detected only in the soil samples, accounting for 24.7% of the classified reads in sample EZS_A and 0.6% in sample AVS_B. The genus Preussia was well-represented in sample AVS_B (17.9%) and showed lower proportions in sample AVS_A (0.7%), whereas the genera Pyrenochaeta, Pleiochaeta, and Dimorphosporicola were present above 0.5% only in sample AVS_B, accounting for 5.1%, 1.4%, and 0.8%, respectively. Finally, the genus Darksidea was detected in low abundance in samples AVS_B (1.5%) and AVS_A (0.9%); the genus Stagonosporopsis accounted for 0.5% in sample AVS_B, while the genus Stemphylium was present in all soil samples, although with an abundance above 0.5% only in AVS_B. Within the order Pezizales, which was present in all soil samples (**Figure 2B**) and dominant in AVS_A and EZS_A (52% and 61%, respectively), the genus Iodophanus was absolutely dominant in sample AVS_A (**Figure 3**), accounting for 46.6% of the total classified reads, but was also detected in samples EZS_D and AVS_B with lower proportions (1.7% and 0.8%). The genus Tricharina was present only in sample EZS_A, in which it constituted 60% of the entire fungal community. Less represented taxa in the Penzizales were the genus Oedocephalum (1.5% in EZS_D and 1.1% in EZS_A) and the genus Ascobolus (0.9% in AVS_A). The order Chaetothyriales was detected in all samples (**Figure 2B**) with an abundance of 1%–4% (in EZS_A below 0.5%) and was significantly represented only by the genus Knufia (**Figure 3**), which was present in all soil samples (0.9%–3.7%) and in the rock sample (1.7%). The order Teloschistales (**Figure 2B**) was present in low proportions in the soil samples AVS_B (1%) and EZS_D (0.9%), but was dominant in the rock sample EZ_D, accounting for 55% of the total classified reads. Within the Teloschistales, the only genus that was found to be significantly abundant was Flavoplaca (**Figure 3**), being clearly dominant in the rock sample (41.5% of the classified reads) and accounting for 0.5% or lower in the soil samples. In EZ_D, 13.7% of the unidentified reads at the genus level belonged to this order. The order Mycosphaerellales was present with lower abundance in two soil samples (0.5% in AVS_A and 4.8% in AVS_B) and higher abundance in the rock sample (EZ_D), accounting for 10% of the total classified reads (**Figure 2B**). This order was significantly represented only by the genus Vermiconidia, which was detected in all samples (except EZS_A), with low proportions in the soil accounting for more than 0.5% only in AVS_B (1.5%) and higher proportions in the rock sample EZ_D, accounting for 9.9% of the total classified reads (**Figure 3**). The order Verrucariales was dominant in sample EZS_D (44%), and it represented only 0.7% of sample AVS_B. Within the Verrucariales, three genera were detected, namely, Catapyrenium, Verrucaria, and Willeya, being mainly present in sample EZS_D, in which Verrucaria was the dominant genus, making up 35.8% of the total fungal community, while Catapyrenium and Willeya were present in low proportions (1.8% and 0.9%, respectively). The order Pertusariales (**Figure 2B**) was solely detected in sample AVS_B and represented only by the genus Circinaria (13.3%), while other orders such as the Candelariales, Saccharomycetales, and Lichinales were exclusively present in sample EZS_D and represented in low proportions by the genera Candelariella (1.5%), Pichia (0.6%), and Gloeoheppia (0.8%), respectively (**Figure 3**). The order Capnodiales was detected only in the rock sample (EZ_D), accounting for 29.8% of the fungal community (**Figure 2B**), but within this order, no taxa could be identified at lower taxonomic levels, therefore, all reads were affiliated with Capnodiales sp. The order Cladosporiales was detected above 0.5% only in sample AVS_B (0.5%), with no genera accounting for more than 0.5%. Finally, within the phylum Ascomycota, the order Hypocreales was also detected in sample EZS_D (0.7%), but no genera belonging to this order were identified above 0.5%.

The phylum Basidiomycota (**Figure 2A**) was mainly represented by the order Agaricales, accounting for 1.2% in EZS_A and 2.8% in EZS_D. Within this order, the genera Arrhenia and Lichenomphalia (sample EZS_D) account for 1.6% and 0.7%, respectively, while the genus Laccaria (sample EZS_A) accounts for 1.1% (**Figure 3**). The order Thelephorales was only represented by the genus Odontia in sample EZS_D at 0.6%, and no genus above 0.5% was identified within the orders Filobasidiales (0.5% in AVS_A) and Cantharellales (0.6% in AVS_B).

The phylum Chytridiomycota (**Figure 2A**), detected in low abundance in the soil samples, was represented by the genus Rhizophlyctis (order Rhizophlyctidales) in sample AVS_B at 0.9%, and by the genus Chytridium (order Chytridiales) in sample EZS_A at 0.7% (**Figure 3**). Finally, the phylum Mucoromycota was only represented by the genus Mortierella (order Mortierellales), with a relatively high abundance of 6% in sample AVS_A (**Figure 3**).

## 4 Discussion

The combined use of culture-dependent and culture-independent approaches enabled us to gain a deeper understanding of the biodiversity of fungal communities associated with the Negev Desert petroglyphs (rock) and their surrounding environment (soil and air).

Both the metagenomic (targeted ITS long-amplicon sequencing) and cultivation studies seem to indicate that the presence of fungi on the stone samples is very limited. In fact, in the metagenomic study, after processing the raw reads, in three out of four rock samples (AV_A, AV_B, and EZ_A), almost no sequences with the correct ITS length (300–900 bp) were present (**Supplementary Table 2**). Since no macroscopic or microscopic evidence of fungi was found in these samples, and the same DNA extraction procedure and PCR primers of the other samples were used, we assume that fungi were in such low proportion to be under the detection limit. The short fragments that were amplified in these samples (**Supplementary Table 2**) are probably just primers and primer-dimers due to the very low template DNA concentration. The only sample in which ITS sequences have been successfully detected was the rock sample without the black varnish, EZ_D. Consistently, the highest amount of cultivable fungi was also recovered from the same sample, while no fungi could be cultivated from the other stone samples (except three colonies from AV_B). The low abundance of fungi in the microbiomes of rocks coated by desert varnish collected from petroglyph sites agrees with previous studies (Lang-Yona et al., 2018; Nir et al., 2021; Rabbachin et al., 2022). The comparison of the data obtained from the soil and rock samples by the culture-based approach and the culture-independent method showed that, as expected, the culture-dependent approach underestimates the total fungal diversity (58 genera were detected using nanopore sequencing (**Supplementary Figure 3**), while 12 were retrieved by cultivation (**Supplementary Table 1**); of these, 9 genera were detected by both techniques). In fact, although culture-based methods are highly valuable in biodiversity studies, they come with limitations and biases (Jeewon and Hyde, 2007), such as the different growth capabilities of fungal taxa under specific experimental conditions. This can lead, for example, to an overestimation of heavily sporulating species and, conversely, to the loss of fungi that are not cultivable or require specific growth conditions (Hamm et al., 2020). On the other end, molecular approaches present the drawback of detecting not only active or dormant members of a microbial community but also dead organisms (Grishkan et al., 2023).

While the soil showed quite high biodiversity (discussed later in this section), the rock mycobiome, according to the metagenomic study, was mostly represented by three genera: *Flavoplaca* (41.5%), *Vermiconidia* (9.9%), and *Knufia* (1.7%) (**Figure 3**). In addition, almost 30% of the fungal community of the stone sample was composed of *Capnodiales* sp. (**Supplementary Figure 4**), which could not be identified at lower taxonomic levels and may belong to an unclassified species. From the cultivation approach, *Alternaria* sp. (which was also detected by the metagenomic analyses in low abundance, 0.3%, **Supplementary Figure 3**), *Cladosporium* sp., *Coniosporium* sp., and one unidentified MCF strain were isolated. The dominant genus *Flavoplaca* (order *Teloschistales*), with the main species detected in this study *Flavoplaca arcis* [basionym: *Caloplaca citrina* var. *arcis* (Arup et al., 2013)] (**Supplementary Figure 4**), is a crustose, saxicolous (rock-dwelling) lichen occurring on limestone (Fletcher and Laundon, 2009), and seems to be commonly present in the Negev Desert (Temina, 2021). Other species identified in this study within the genera *Vermiconidia*, *Knufia*, and *Coniosporium*, as well as *Phaeotheca* and *Devriesia*, were also identified on the stone sample, but in lower proportions (0.4% and 0.2%, respectively, **Supplementary Figure 3**). These fungi are all microcolonial fungi (Isola et al., 2016; Liu B. et al., 2022). According to Isola et al (Isola et al., 2016), in natural, harsh environments, rock fungi in the class *Dothideomycetes*, predominate over MCF belonging to the order *Chaetothyriales*, and this is in accordance with our results. Indeed, except the genus *Knufia* (order *Chaetothyriales*, class *Eurotyomycetes*), the other MCF identified on the stone in this study belong to the class *Dothideomycetes*, in which many species exhibit adaptation mechanisms to extreme conditions, such as thick melanized cellular walls, meristematic development, morphological and metabolic versatility, mycosporine-like substances, and oligotrophy (Gorbushina et al., 2003; Gostinĉar et al., 2012; Zakharova et al., 2013; De Leo et al., 2022). The ability of MCF to weather stone, which occurs through both mechanical (inter-crystalline growth) and chemical (release of organic acids) processes, poses a threat to stone cultural heritage, such as the petroglyphs of the Negev Desert (De Leo et al., 2022). For example, the species *Vermiconidia calcicola*, also identified in this study (5.8% of the fungal community of the stone sample EZ_D) (**Supplementary Figure 4**), and *Coniosporium* sp. (isolated from sample EZ_D) (**Table 1**) have been studied in relation to the biodeterioration of marble, and their ability to release acids and dissolve calcium carbonate (on CaCO_3_ agar medium) has been demonstrated (Berti et al., 2023). Favero-Longo et al. (2011) hypothesized instead that MCF dissolves calcium carbonate through the production of siderophores. In this study, we tested the ability of the isolated strains from soil and stone to dissolve calcium carbonate through acid production in laboratory settings. Unfortunately, all tested strains, including *Coniosporium* sp. (strain NS14, **Table 1**), resulted in negative results, and only strain NS16, *Preussia australis*, isolated from soil, resulted in positive results (**Supplementary Figure 2**). This species, together with *Sporormiella* spp. (Preussia/Sporormiella species complex), occurred frequently in the soil (**Table 1**; **Figure 3**), and, indeed, they have been often reported as fungal endophytes in deserts and arid environments (Massimo et al., 2015; González-Menéndez et al., 2018; Sandberg et al., 2022). Preussia/Sporomiella species have the ability to produce a variety of bioactive secondary metabolites with antibacterial, antifungal, and antitumoral activities (Mapperson et al., 2014) (González-Menéndez et al., 2018). Within these secondary metabolites, there are also some organic acids, which, in this case, might have induced the dissolution of the calcium carbonate in the plate assays.

**Figure 4:**
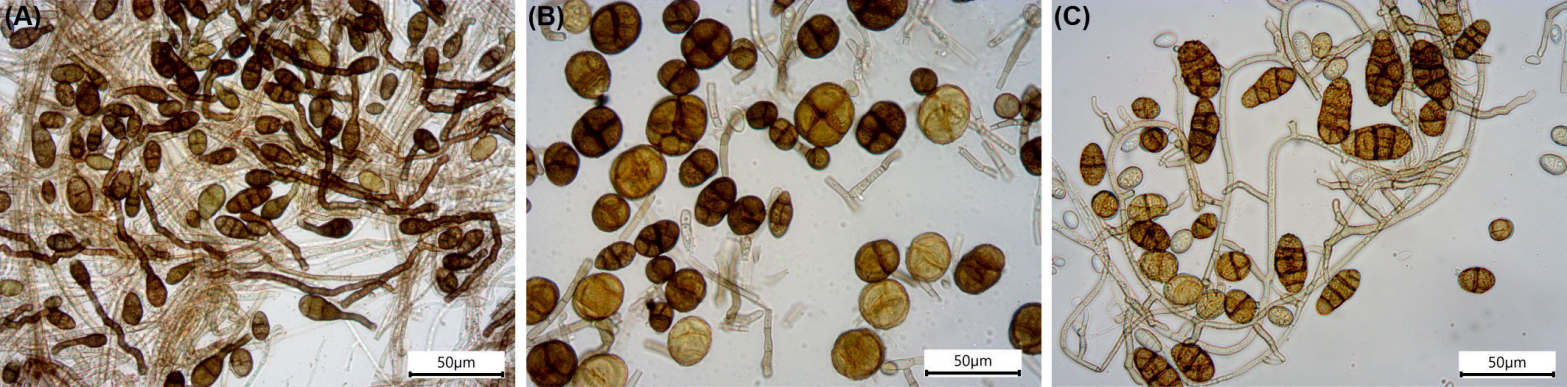
Microscopy pictures of *Alternaria* spp. conidia isolated from the Negev Desert topsoil. **(A)** Strain NS10, *Alternaria* sp. in A. sect. *Alternaria*; **(B)** strain NS1, *Alternaria* sp. in *A. sect. Ulocladioides*; and **(C)** strain NS4, *Alternaria* sp. in *A. sect. Ulocladium*.

Nevertheless, in the soil mycobiome, the genus *Alternaria* was the only one consistently present in all samples and consistently detected by both metagenomic and culture-based approaches. Not only was it found in high abundance (**Figure 3**; **Table 1**), but it also exhibited the highest biodiversity. Seven species were identified by nanopore sequencing (**Supplementary Figure 4**), and 13 strains (NS1–NS13) belonging to seven different *Alternaria* sections were retrieved by cultivation (**Table 1**). The abundant presence of *Alternaria* in the Negev topsoil aligns perfectly with previous results obtained by Griskhan and colleagues (Yu et al., 2012; Grishkan and Kidron, 2013; Grishkan et al., 2023) and with other biodiversity studies on desert soil (Conley et al., 2006; Bates et al., 2012; Zhang et al., 2016). *Alternaria* species exhibited strongly melanized, large, thick-walled, and multicellular spores (examples are shown in **Figure 4**), all characteristics that enable these fungi to thrive in high temperatures, strong UV radiation, and xeric conditions (Grishkan, 2019). In our study, the species *A. chlamydosporigena*, *A. alternata*, and A. *chlamydospora* were the most abundant based on the metagenomic results (**Supplementary Figure 4**), consistent with the findings by Grishkan et al. (2023). Additionally, one-third of the cultivable *Alternaria* spp. belong to the *Alternaria* sect. *Ulocladioides* and *Alternaria* sect. *Ulocladium*, both known for their dark colonies (**Supplementary Figures 1E, F**) with very large, extremely resistant spores (**Figures 4B, C**).

The culture-based approach showed that *Cladosporium* spp. occurred frequently in the soil (**Table 1**), while metagenomic analyses detected this genus in low abundance (< 0.5%, **Supplementary Figure 3**). *Cladosporium* is a common and ubiquitous group of hyphomycetes in the environment, capable of adapting to extreme temperatures and xeric conditions by developing thick-walled, melanized conidia capable of rehydration and germination within hours (Abdollahzadeh et al., 2020; Peng et al., 2021). The presence of *Cladosporium*, particularly species from the *C. cladosporioides* complex (strains NS20, NA1, NA2, NA3, and NA16; **Table 1**), as a common fungal taxon in desert soil is not surprising and has been observed by other authors (Santiago et al., 2018; Ameen et al., 2022; Grishkan et al., 2023). Additionally, *Cladosporium* is one of the most common airborne fungi (De Antoni Zoppas et al., 2006; Herrero et al., 2006; Bensch et al., 2012; Anees-Hill et al., 2022) and has been reported as a major component of the air mycobiota in desert environments (Ismail et al., 2002). In our study, *Cladosporium* spp. were the most abundant strains retrieved from the air sampling (**Table 1**). Interestingly, species within the *C. sphaerospermum* complex (strains NS19, NA12, and NA13; **Table 1**), although common cosmopolitan airborne species, are often associated with extreme environments, such as hypersaline locations (Zalar et al., 2007; Sterflinger et al., 2012), and strains identified as *C. sphaerospermum* have been shown to grow at very low water activity (as low as 0.815) (Hocking et al., 1994; Gostinčar et al., 2010). From the air sampling, *Nigrospora* and *Stemphylium* strains were also isolated; both are melanized fungi producing large spores (Grishkan et al., 2023). *Stemphylium* was also present in all soil samples, according to the metagenomic analysis, although in low abundance (**Supplementary Figure 3**).

In the four soil samples, four dominant genera were identified solely through metagenomic analysis. These genera, *Iodophanus* (46.6% of sample AVS_A), *Circinaria* (13.3% of sample AVS_B), *Tricharina* (60.1% of sample EZS_A), and *Verrucaria* (35.8% of sample EZS_D), were detected solely by the metagenomic analysis (**Figure 3**). The genus *Tricharina* (order *Pezizales*) was previously identified as part of the microbiome associated with the rhizosphere of plants in the Atacama Desert (Fuentes et al., 2020), while the genus *Iodophanus* (order *Pezizales*), whose members are rich in carotenoid pigments, was found in desert soil by a few studies (Abed et al., 2010; Ameen et al., 2021; Wang et al., 2022). The genera *Circinaria* and *Verrucaria*, together with the genus *Flavoplaca*, detected on the stone sample, are instead lichen-forming fungi (Miadlikowska et al., 2006; Thüs et al., 2011) and therefore cannot be isolated under laboratory conditions. In lower abundance in the soil samples, other lichen-forming species were also identified, such as *Catapyrenium* sp., *Willeya* sp., *Candelariella* spp., and *Gloeoheppia turgida* (**Supplementary Figure 4**). Most lichens in the Negev Desert are saxicolous species that colonize limestone substrates (Temina, 2021). In this study, except for *Catapyrenium* and *Gloeoheppia*, the other lichens identified belong to this category.

As for MCF, lichens are able to withstand extreme conditions (Armstrong, 2017; Gostinčar et al., 2023), they are rock specialists, and are well-known stone deteriogens (e.g. 494 Salvadori and Casanova, 2016; Cozzolino et al., 2022). A few studies report serious damage to numerous rock art sites due to lichen colonization (Knight et al., 2004; Dandridge, 2006). On the other hand, lichens, along with other biofilms, can have a dual role and, in some cases, can be considered protectants of stone monuments (Pinna, 2014) (Mcllroy de la Rosa et al., 2013) (Favero-Longo and Viles, 2020). In fact, research has shown that lichens have bioprotective effects through physical shielding, which can mitigate the impact of abiotic weathering (e.g., rain, wind, thermal stress) on the rock, or through the formation of an insoluble crust, typically calcium oxalate, at the lichen-rock interface, thereby safeguarding the underlying stone (Mcllroy de la Rosa et al., 2013). (Pinna, 2014). Nevertheless, in order to assess whether a bioprotective or biodeteriorative action is prevailing, both the abiotic and biogenic weathering processes should be properly weighed and evaluated case by case (Liu X. et al., 2022). In the context of this study, we believe that biodeteriorative effects are prevailing. Indeed, while, for example, in a humid and rainy environment, limestone dissolution rates are high, thus a lichen cover might protect the stone substrate, in a dry environment such as the Negev Desert, where dissolution rates are extremely low, lichen-induced surface loss may exceed surface loss from dissolution, leading to biodeterioration (Mcllroy de la Rosa et al., 2013). Moreover, at the Negev Desert rock art sites, some stones are visibly colonized by epilithic lichens, obscuring the black rock surface on which the petroglyphs are carved and causing an unacceptable aesthetic impairment.

In a recent study on the biodeterioration of Negev petroglyph sites, Nir et al. (2021) suggested that lichens play a role in both the deterioration of the bedrock (calcium carbonate) and the disintegration of the external desert varnish. They observed that in samples colonized by lichens, the black layer was no longer present. Additionally, lichens can accelerate the weathering of the desert varnish, which is composed of clay minerals and amorphous silica in a matrix of manganese and iron oxides, through mineral dissolution of manganese and iron oxides (Nir et al., 2021).

Finally, the molecular approach also allowed the detection of other phyla in the soil samples, namely, *Basidiomycota*, *Chytridiomycota*, and *Mucoromycota* (**Figure 2A**). While *Basydiomycota* taxa, such as *Arrhenia*, *Lichenomphalia*, a lichenized agaric genus (Sandoval-Leiva et al., 2017), and *Laccaria* (**Figure 3**) are rarely found in desert environments, members of *Chytridiomycota*, such as *Rhizophlyctis* spp., and members of *Mucoromycota*, such as *Mortierella* spp., were previously identified in desert soil (Murgia et al., 2018; Grishkan et al., 2023).

## 5 Conclusions

The Negev Desert soil showed high fungal biodiversity, formed by many cosmopolitan species that are resistant to harsh environmental conditions due to, for example, the production of melanized, large, thick-walled spores (**Figure 4**). Our results indicate that the rock mycobiota, if detectable, is instead highly specialized and consists of extremotolerant fungal species known to deteriorate limestone (MCF and lichens). The comparison of the rock mycobiota to the fungal communities in the environment (soil and air) clearly shows that Cladosporium and Alternaria, detected in low abundance on the stone sample, as well as other cosmopolitan species previously found on those petroglyphs (Rabbachin et al., 2022), come from the surrounding environment and should be regarded as transient. The three stone samples presenting the black varnish (AV_A, AV_B, and EZ_B) show no apparent fungal colonization, or if present, fungi are below the detection limit of the techniques used in this study. On the other hand, on the stone sample without the black crust (EZ_D), fungi were detected both by ITS sequencing and cultivation. Based on this, together with results from previous studies including microscopical and element analysis (Nir et al., 2021; Rabbachin et al., 2022), we can conclude that in this sample, the exfoliation of the black layer has been enhanced by the detected highly biodeteriorative fungi.

## Data availability statement

The datasets presented in this study can be found in online repositories. The names of the repository/repositories and accession number(s) can be found in the article/**Supplementary Material**.

## Author contributions

LR: Conceptualization, Data curation, Investigation, Visualization, Writing – original draft, Writing – review & editing. IN: Data curation, Investigation, Resources, Writing – review & editing. MW: Data curation, Formal Analysis, Visualization, Writing – review & editing. YV: Investigation, Writing – review & editing. GP: Conceptualization, Supervision, Writing – review & editing. AG: Supervision, Writing – review & editing. AK: Supervision, Writing – review & editing. KS: Conceptualization, Supervision, Writing – review & editing.

## References

[B1] Abdollahzadeh J. Groenewald J. Z. Coetzee M. P.A. Wingfield M. J. Crous P. W. (2020). Evolution of lifestyles in capnodiales. Stud. Mycology 95, 381–414. doi: 10.1016/j.simyco.2020.02.004 PMC7426231 32855743

[B2] Abed R. M. M. Al Kharusi S. Schramm A. Robinson M. D. (2010). Bacterial diversity, pigments and nitrogen fixation of biological desert crusts from the Sultanate of Oman. FEMS Microbiol. Ecol. 72, 418–428. doi: 10.1111/fem.2010.72.issue-3 20298501

[B3] Albertano P. Urzí C. (1999). Structural interactions among epilithic cyanobacteria and heterotrophic microorganisms in roman hypogea. Microbial Ecol. 38, 244–252. doi: 10.1007/s002489900174 10541786

[B4] Ameen F. Stephenson S. L. Al Nadhari S. Yassin M. A. (2021). A review of fungi associated with arabian desert soils. Nova Hedwigia 112, 173–195. doi: 10.1127/nova_hedwigia/2020/0611

[B5] Ameen F. AlNAdhari S. Yassin M. A. Al-Sabri A. Almansob A. Alqahtani N.. (2022). Desert soil fungi isolated from Saudi Arabia: cultivable fungal community and biochemical production. Saudi J. Biol. Sci. 29, 2409–2420. doi: 10.1016/j.sjbs.2021.12.011 35531195 PMC9072920

[B6] Andreae M. O. Al-Amri A. Andreae T. W. Garfinkel A. Haug G. Jochum K. P.. (2020). Geochemical studies on rock varnish and petroglyphs in the Owens and Rose Valleys, California. PLoS One 15, 1–32. doi: 10.1371/journal.pone.0235421 PMC7405993 32756552

[B7] Anees-Hill S. Douglas P. Pashley C. H. Hansell A. Marczylo E. L. (2022). A systematic review of outdoor airborne fungal spore seasonality across Europe and the implications for health. Sci. Total Environ. 818, 151716. doi: 10.1016/j.scitotenv.2021.151716 34800445 PMC8919338

[B8] Armstrong R. (2017). “Adaptation of lichens to extreme conditions” in Plant Adaptation Strategies in Changing Environment. Eds. Shukla V. Kumar S. Kumar N. (Springer, Singapore). doi: 10.1007/978-981-10-6744-0_1

[B9] Arup U. Søchting U. Frödén P. (2013). A new taxonomy of the family Teloschistaceae. Nordic J. Bot. 31, 016–083. doi: 10.1111/j.1756-1051.2013.00062.x

[B10] Bates S. T. Nash T. H. III Garcia-Pichel F. (2012). Patterns of diversity for fungal assemblages of biological soil crusts from the southwestern United States. Mycologia 104, 353–361. doi: 10.3852/11-232 22123652

[B11] Bensch K. Braun U. Groenewald J. Z. Crous P. W. (2012). The genus cladosporium. Stud. Mycology 72, 1–401. doi: 10.3114/sim0003 PMC3390897 22815589

[B12] Berti L. Marvasi M. Perito B. (2023). Characterization of the community of black meristematic fungi inhabiting the external white marble of the florence cathedral. J. Fungi 9, 665. doi: 10.3390/jof9060665 PMC10301995 37367601

[B13] Chen A. (2017). Spatially explicit modelling of agricultural dynamics in semi-arid environments. Ecol. Model. 363, 31–47. doi: 10.1016/j.ecolmodel.2017.08.025

[B14] Coleine C. Stajich J. E. de los Ríos A. Selbmann L. (2021). Beyond the extremes: Rocks as ultimate refuge for fungi in drylands. Mycologia 113, 108–133. doi: 10.1080/00275514.2020.1816761 33232202

[B15] Coleine C. Delgado-Baquerizo M. Albanese D. Singh B. K. Stajich J. E. Selbmann L.. (2022). Rocks support a distinctive and consistent mycobiome across contrasting dry regions of Earth’. FEMS Microbiol. Ecol. 113, 108–133. doi: 10.1093/femsec/fiac030 35298630

[B16] Conley C. A. Ishkhanova G. Mckay C. P. Cullings K. (2006). A preliminary survey of non-lichenized fungi cultured from the hyperarid Atacama Desert of Chile. Astrobiology 6, 521–526. doi: 10.1089/ast.2006.6.521 16916279

[B17] Cozzolino A. Adamo P. Bonanomi G. Motti R. (2022). The role of lichens, mosses, and vascular plants in the biodeterioration of historic buildings: A review. Plants 11, 1–19. doi: 10.3390/plants11243429 PMC9781475 36559541

[B18] Dandridge D. E. (2006). Lichens: The Challenge for Rock Art Conservation (Texas A & M University).

[B19] De Antoni Zoppas B. C. Valencia-Barrera R. M. Vergamini Duso S. M. Fernández-González D. (2006). Fungal spores prevalent in the aerosol of the city of Caxias do Sul, Rio Grande do Sul, Brazil, over a 2-year period, (2001–2002). Aerobiologia 22, 117–124. doi: 10.1007/s10453-006-9022-2

[B20] De Leo F. Marchetta A. Urzì C. (2022). Black fungi on stone-built heritage: Current knowledge and future outlook. Appl. Sci. (Switzerland) 12, 3969. doi: 10.3390/app12083969

[B21] de Los Ríos A. Cámara B. García del Cura M. Á. Rico V. J. Galván V. Ascaso C. (2009). Deteriorating effects of lichen and microbial colonization of carbonate building rocks in the Romanesque churches of Segovia, Spain. Sci. total Environ. 407, 1123–1134. doi: 10.1016/j.scitotenv.2008.09.042 18976800

[B22] Eisenberg-Degen D. Frumkin R. Gur D. Horwitz L. K. Nash G. Rosen S. A.. (2022). Rocks of Ages : Developing Rock Art Tourism in Israel. Ed. Schmidt J. (Oxford UK: Archaeopress Publishing). doi: 10.2307/j.ctv2bz2mhn

[B23] Eisenberg-Degen D. Nash G. (2014). Hunting and gender as reflected in the central negev rock art, Israel. Time Mind 7, 259–277. doi: 10.1080/1751696X.2014.956009

[B24] Favero-Longo S. E. Gazzano C. Girlanda M. Castelli D. Tretiach M. Baiocchi C.. (2011). Physical and chemical deterioration of silicate and carbonate rocks by meristematic microcolonial fungi and endolithic lichens (Chaetothyriomycetidae). Geomicrobiology J. 28, 732–744. doi: 10.1080/01490451.2010.517696

[B25] Favero-Longo S. E. Viles H. A. (2020). A review of the nature, role and control of lithobionts on stone cultural heritage: weighing-up and managing biodeterioration and bioprotection. World J. Microbiol. Biotechnol. 36, 1–18. doi: 10.1007/s11274-020-02878-3 32607867

[B26] Fletcher A. Laundon J. R. (2009). “Caloplaca” in Lichens of Great Britain & Ireland (British Lichen Society, London), 245–273.

[B27] Fuentes A. Herrera H. Charles T. C. Arriagada C. (2020). Fungal and bacterial microbiome associated with the rhizosphere of native plants from the atacama desert. Microorganisms 8, 209. doi: 10.3390/microorganisms8020209 32033093 PMC7074712

[B28] Gómez-Silva B. Vilo-Muñoz C. Galetovic A. Dong Q. Castelán-Sánchez H. G. Pérez-Llano Y.. (2019). Metagenomics of atacama lithobiontic extremophile life unveils highlights on fungal communities, biogeochemical cycles and carbohydrate-active enzymes. Microorganisms 7, 619. doi: 10.3390/microorganisms7120619 31783517 PMC6956184

[B29] Gonçalves V. N. Cantrell C. L. Wedge D. E. Ferreira M. C. Soares M. A. Jacob M. R.. (2016). Fungi associated with rocks of the Atacama Desert: Taxonomy, distribution, diversity, ecology and bioprospection for bioactive compounds. Environ. Microbiol. 18, 232–245. doi: 10.1111/1462-2920.13005 26235221

[B30] González-Menéndez V. Crespo G. de Pedro N. Diaz C. Martín J. Serrano R.. (2018). Fungal endophytes from arid areas of Andalusia: High potential sources for antifungal and antitumoral agents. Sci. Rep. 8, 1–13. doi: 10.1038/s41598-018-28192-5 29950656 PMC6021435

[B31] Gorbushina A. A. Whitehead K. Dornieden T. Niesse A. Schulte A. Hedges J. I. (2003). Black fungal colonies as units of survival: Hyphal mycosporines synthesized by rock-dwelling microcolonial fungi. Can. J. Bot. 81, 131–138. doi: 10.1139/b03-011

[B32] Gostinčar C. Grube M. De Hoog S. Zalar P. Gunde-Cimerman N. (2010). Extremotolerance in fungi: Evolution on the edge. FEMS Microbiol. Ecol. 71, 2–11. doi: 10.1111/fem.2010.71.issue-1 19878320

[B33] Gostinĉar C. Muggia L. Grube M. (2012). Polyextremotolerant black fungi: Oligotrophism, adaptive potential, and a link to lichen symbioses. Front. Microbiol. 3. doi: 10.3389/fmicb.2012.00390 PMC3492852 23162543

[B34] Gostinčar C. Stajich J. E. Gunde-Cimerman N. (2023). ‘Extremophilic and extremotolerant fungi’. Curr. Biol. 33, R752–R756. doi: 10.1016/j.cub.2023.06.011 37490857

[B35] Grishkan I. (2019). “Soil microfungi of Israeli deserts: adaptations to environmental stress” in Fungi in extreme environments: ecological role and biotechnological significance. Eds. Tiquia-Arashiro S. Grube M. (Springer Nature, Switzerland AG), 97–117. doi: 10.1007/978-3-030-19030-9

[B36] Grishkan I. Kidron G. J. Rodriguez-Berbel N. Miralles I Ortega R. (2023). Altitudinal gradient and soil depth as sources of variations in fungal communities revealed by culture-dependent and culture-independent methods in the negev desert, Israel. Microorganisms 11, 1761. doi: 10.3390/microorganisms11071761 37512933 PMC10383159

[B37] Grishkan I. Kidron G. J. (2013). Biocrust-inhabiting cultured microfungi along a dune catena in the western Negev Desert, Israel. Eur. J. Soil Biol. 56, 107–114. doi: 10.1016/j.ejsobi.2013.03.005

[B38] Grishkan I. Temina M. (2023). Composition and diversity of endolichenic microfungal communities from saxicolous lichens at Nahal Boker, the central Negev Desert, Israel. Fungal Ecol. 61, 101196. doi: 10.1016/j.funeco.2022.101196

[B39] Hamm P. S. Mueller R. C. Kuske C. R. Porras-Alfaro A. (2020). Keratinophilic fungi: Specialized fungal communities in a desert ecosystem identified using cultured-based and Illumina sequencing approaches. Microbiological Res. 239, 126530. doi: 10.1016/j.micres.2020.126530 32622287

[B40] Herrero A. D. Sabariego Ruiz S. Gutiérrez Bustillo M. Cervigón Morales P. (2006). Study of airborne fungal spores in Madrid, Spain. Aerobiologia 22, 135–142. doi: 10.1007/s10453-006-9025-z

[B41] Hocking A. D. Miscamble B. F. Pitt J. I. (1994). Water relations of Alternaria alternata, Cladosporium cladosporioides, Cladosporium sphaerospermum, Curvularia lunata and Curvularia pallescens. Mycological Res. 98, 91–94. doi: 10.1016/S0953-7562(09)80344-4

[B42] Ismail M. A. Abdel-Hafez S. I. I. Moharram A. M. (2002). Aeromycobiota of Western Desert of Egypt. Afr. J. Sci. Technol. 3. doi: 10.4314/ajst.v3i1.15280

[B43] Isola D. Zucconi L. Onofri S. Caneva G. de Hoog G. S. Selbmann L. (2016). Extremotolerant rock inhabiting black fungi from Italian monumental sites. Fungal Diversity 76, 75–96. doi: 10.1007/s13225-015-0342-9

[B44] Israel Meteorological service (2024). Available at: https://ims.gov.il/en/ClimateAtlas.

[B45] Jeewon R. Hyde K. D. (2007). ‘Detection and diversity of fungi from environmental samples: Traditional versus molecular approaches’. Advance techniques in soil microbiology. 11, 1–15. doi: 10.1007/978-3-540-70865-0_1

[B46] Knight K. St. Clair L. Gardner J. (2004). “Lichen biodeterioration at inscription rock, el morro national monument, ramah, new Mexico, USA” in Biodeterioration of Stone Surfaces. Eds. St.Clair L. L. Seaward M. R. D. (Berlin, Germany: Springer), 129–163.

[B47] Krumbein W. E. Jens K. (1981). Biogenic rock varnishes of the negev desert (Israel) an ecological study of iron and manganese transformation by cyanobacteria and fungi. Oecologia 50, 25–38. doi: 10.1007/BF00378791 28310059

[B48] Lang-Yona N. Maier S. Macholdt D. S. Müller-Germann I. Yordanova P. Rodriguez-Caballero E.. (2018). Insights into microbial involvement in desert varnish formation retrieved from metagenomic analysis. Environ. Microbiol. Rep. 10, 264–271. doi: 10.1111/1758-2229.12634 29488349

[B49] Liu B. Fua R. Wu B. Liu X. Xiang M. (2022). Rock-inhabiting fungi: terminology, diversity, evolution and adaptation mechanisms. Mycology 13, 1–31. doi: 10.1080/21501203.2021.2002452 35186410 PMC8856086

[B50] Liu X. Qian Y. Wu F. Wang Y. Wang W. Gu J.-D. (2022). Biofilms on stone monuments: biodeterioration or bioprotection? Trends Microbiol. 30, 816–819. doi: 10.1016/j.tim.2022.05.012 35752563

[B51] Mapperson R. R. Kotiw M. Davis R. A. Dearnaley J. D. W. (2014). The diversity and antimicrobial activity of preussia sp. Endophytes isolated from Australian dry rainforests. Curr. Microbiol. 68, 30–37. doi: 10.1007/s00284-013-0415-5 23975673

[B52] Marvasi M. Donnarumma F. Frandi A. Mastromei G. Sterflinger K. Tiano P.. (2012). Black microcolonial fungi as deteriogens of two famous marble statues in Florence, Italy. Int. Biodeterioration Biodegradation 68, 36–44. doi: 10.1016/j.ibiod.2011.10.011

[B53] Massimo N. C. Nandi Devan M. M. Arendt K. R. Wilch M. H. Riddle J. M. Furr S. H.. (2015). Fungal endophytes in aboveground tissues of desert plants: Infrequent in culture, but highly diverse and distinctive symbionts. Microbial Ecol. 70, 61–76. doi: 10.1007/s00248-014-0563-6 PMC4457668 25645243

[B54] Mcllroy de la Rosa J. P. Warke P. A. Smith B. J. (2013). Lichen-induced biomodification of calcareous surfaces: Bioprotection versus biodeterioration. Prog. Phys. Geogr. 37, 325–351. doi: 10.1177/0309133312467660

[B55] Miadlikowska J. Kauff F. Hofstetter V. Fraker E. Hafellner J. Reeb V.. (2006). New insights into classification and evolution of the Lecanoromycetes (Pezizomycotina, Ascomycota) from phylogenetic analyses of three ribosomal RNA- and two protein-coding genes. Mycologia 98, 1088–1103. doi: 10.1080/15572536.2006.11832636 17486983

[B56] Murgia M. Fiamma M. Barac A. Deligios M. Mazzarello V. Paglietti B.. (2018). Biodiversity of fungi in hot desert sands. MicrobiologyOpen 8. doi: 10.1002/mbo3.595 PMC6341031 29504263

[B57] Nir I. Barak H. Kramarsky-Winter E. Kushmaro A. (2019). Seasonal diversity of the bacterial communities associated with petroglyphs sites from the Negev Desert, Israel. Ann. Microbiol. 69, 1079–1086. doi: 10.1007/s13213-019-01509-z

[B58] Nir I. Barak H. Kramarsky-Winter E. Kushmaro A. de los Ríos A. (2021). Microscopic and biomolecular complementary approaches to characterize bioweathering processes at petroglyph sites from the Negev Desert, Israel. Environ. Microbiol. 24, 967–980. doi: 10.1111/1462-2920.15635 34110072

[B59] Nir I. Hana B. Laura R. Arielle K. Mariela P. Esti K.-W.. (2023). ‘Trichocoleus desertorum isolated from Negev desert petroglyphs: Characterization, adaptation and bioerosion potential’. Sci. Total Environ. 904. doi: 10.1016/j.scitotenv.2023.166739 37673239

[B60] Onofri S. Zucconi L. Isola D. Selbmann L. (2014). Rock-inhabiting fungi and their role in deterioration of stone monuments in the Mediterranean area. Plant Biosyst. 148, 384–391. doi: 10.1080/11263504.2013.877533

[B61] Paiva D. S. Trovão J. Fernandes L. Mesquita N. Tiago I. Portugal A. (2023). Expanding the Microcolonial Black Fungi Aeminiaceae Family: Saxispiralis lemnorum gen. et sp. nov. (Mycosphaerellales), Isolated from Deteriorated Limestone in the Lemos Pantheon, Portugal. J. Fungi 9, 916. doi: 10.3390/jof9090916 PMC10533162 37755024

[B62] Pangallo D. Kraková L. Chovanová K. Šimonovičová A. De Leo F. Urzì C. (2012). Analysis and comparison of the microflora isolated from fresco surface and from surrounding air environment through molecular and biodegradative assays. World J. Microbiol. Biotechnol. 28, 2015–2027. doi: 10.1007/s11274-012-1004-7 22806023

[B63] Peng X. Gat D. Paytan A. Rudich Y. (2021). The response of airborne mycobiome to dust storms in the eastern mediterranean. J. Fungi 7, 802. doi: 10.3390/jof7100802 PMC8540267 34682226

[B64] Perry R. S. Lynne B. Y. Sephton M. A. Kolb V. M. Perry C. C. Staley J. T. (2006). Baking black opal in the desert sun: The importance of silica in desert varnish. Geology 34, 537–540. doi: 10.1130/G22352.1

[B65] Pinna D. (2014). ‘Biofilms and lichens on stone monuments: Do they damage or protect?’. Front. Microbiol. 5. doi: 10.3389/fmicb.2014.00133 PMC3980096 24765088

[B66] Rabbachin L. Piñar G. Nir I. Kushmaro A. Pavan M. J. Eitenberger E.. (2022). A multi-analytical approach to infer mineral–microbial interactions applied to petroglyph sites in the negev desert of Israel. Appl. Sci. (Switzerland) 12, 6936. doi: 10.3390/app12146936

[B67] Salvadori O. Casanova A. (2016). The role of fungi and lichens in the biodeterioration of stone monuments. Open Conf. Proc. J. 10, 29–38. doi: 10.2174/2210289201607020039

[B68] Sandberg D. C. Del Olmo-Ruiz M. Sykes B. E. Woods D. O. Arnold A. E. (2022). Three distinctive Preussia (Sporormiaceae) from photosynthetic stems of Ephedra trifurca (Ephedraceae, Gnetophyta) in southeastern Arizona, USA. Plant Fungal Systematics 67, 63–74. doi: 10.35535/pfsyst-2022-0008

[B69] Sandoval-Leiva P. Niveiro N. Urbina-Casanova R. Scherson R. (2017). Lichenomphalia Altoandina, a new species of hygrophoraceae from the Chilean Altiplano. Mycologia 109, 92–99. doi: 10.1080/00275514.2017.1281682 28402793

[B70] Santiago I. F. Gonçalves V. N. Gómez-Silva B. Galetovic A. Rosa L. H. (2018). Fungal diversity in the atacama desert. Antonie van Leeuwenhoek Int. J. Gen. Mol. Microbiol. 111, 1345–1360. doi: 10.1007/s10482-018-1060-6 29516313

[B71] Selbmann L. Zucconi L. Isola D. Onofri S. (2015). Rock black fungi: excellence in the extremes, from the Antarctic to space. Curr. Genet. 61, 335–345. doi: 10.1007/s00294-014-0457-7 25381156

[B72] Staley J. T. Palmer F. Adams J. (1989). Microcolonial fungi: Common inhabitants on desert rocks? Science 215, 1093–1095. doi: 10.1126/science.215.4536.1093 17771840

[B73] Steiger M. Charola E. Sterflinger K. (2014). “Weathering and deterioration” in Stone in Architecture: Properties, Durability, Fifth Edition (Heidelberg, Germany: Springer), 225–316. doi: 10.1007/978-3-642-45155-3

[B74] Sterflinger K. Krumbein W. E. (1997). Dematiaceous fungi as a major agent for biopitting on mediterranean marbles and limestones. Geomicrobiology J. 14, 219–230. doi: 10.1080/01490459709378045

[B75] Sterflinger K. Tesei D. Zakharova K. (2012). Fungi in hot and cold deserts with particular reference to microcolonial fungi. Fungal Ecol. 5, 453–462. doi: 10.1016/j.funeco.2011.12.007

[B76] Tang Y. Cheng J. Lian B. (2016). Characterization of endolithic culturable microbial communities in carbonate rocks from a typical karst canyon in Guizhou (China). Polish J. Microbiol. 65, 413–423. doi: 10.5604/17331331.1227667 28735325

[B77] Temina M. (2021). “Lichens of the negev desert (Israel): diversity, distribution, and relationship with microclimate” in Biodiversity, Conservation and Sustainability in Asia. Eds. Öztürk M. Altay V. Efe R. (Springer Nature, Switzerland AG), 23–37. doi: 10.1007/978-3-030-59928-7

[B78] Thüs H. Muggia L. Pérez-Ortega S. Favero-Longo S. E. Joneson S. O’Brien H.. (2011). Revisiting photobiont diversity in the lichen family Verrucariaceae (Ascomycota). Eur. J. Phycology 46, 399–415. doi: 10.1080/09670262.2011.629788

[B79] Tichy J. Waldherr M. Ortbauer M. Graf A. Sipek B. Jembrih-Simbuerger D.. (2023). ‘Pretty in pink? Complementary strategies for analysing pink biofilms on historical buildings’. Sci. Total Environ. 904. doi: 10.1016/j.scitotenv.2023.166737 37659529

[B80] Viani I. Colucci M. E. Pergreffi M. Rossi D. Veronesi L. Bizzarro A.. (2020). Passive air sampling: The use of the index of microbial air contamination. Acta Biomedica 91, 92–105. doi: 10.23750/abm.v91i3-S.9434 PMC7975895 32275273

[B81] Wang J. Qu M. Wang Y. He N. Li J. (2022). Plant traits and community composition drive the assembly processes of abundant and rare fungi across deserts. Front. Microbiol. 13. doi: 10.3389/fmicb.2022.996305 PMC9554466 36246243

[B82] White T. J. Bruns T. Lee S. Taylor J. (1990). “Amplification and direct sequencing of fungal ribosomal RNA Genes for phylogenetics” in PCR - Protocols and Applications - A Laboratory Manual (Academic Press), 315–322. doi: 10.1016/B978-0-12-372180-8.50042-1

[B83] Yu J. Grishkan I. Sherman C. Steinberger Y. (2012). Spatiotemporal variability of cultivable microfungal communities inhabiting a playa area in the western Negev Desert, Israel. J. Arid Environments 81, 9–17. doi: 10.1016/j.jaridenv.2012.01.005

[B84] Zakharova K. Tesei D. Marzban G. Dijksterhuis J. Wyatt T. Sterflinger K. (2013). Microcolonial fungi on rocks: A life in constant drought? Mycopathologia 175, 537–547. doi: 10.1007/s11046-012-9592-1 23073825 PMC3669513

[B85] Zalar P. de Hoog G. S. Schroers H.-J. Crous P. W. Groenewald J. Z. Gunde-Cimerman N. (2007). Phylogeny and ecology of the ubiquitous saprobe Cladosporium sphaerospermum, with descriptions of seven new species from hypersaline environments. Stud. Mycology 58, 157–183. doi: 10.3114/sim.2007.58.06 PMC2104741 18490999

[B86] Zhang T. Jia R. L. Yu L. Y. (2016). Diversity and distribution of soil fungal communities associated with biological soil crusts in the southeastern Tengger Desert (China) as revealed by 454 pyrosequencing. Fungal Ecol. 23, 156–163. doi: 10.1016/j.funeco.2016.08.004

