# Supplementary material for: Diversity of fungi associated with petroglyph sites in the Negev Desert, Israel, and their potential role in bioweathering
Source: Front Fungal Biol. 2024 Jul 5;5:1400380. doi: 10.3389/ffunb.2024.1400380 (PMC11257853; doi:10.3389/ffunb.2024.1400380)
Supplement: Supplementary file 1 [file DataSheet_1.docx]

Supplementary Material

# Supplementary Figures


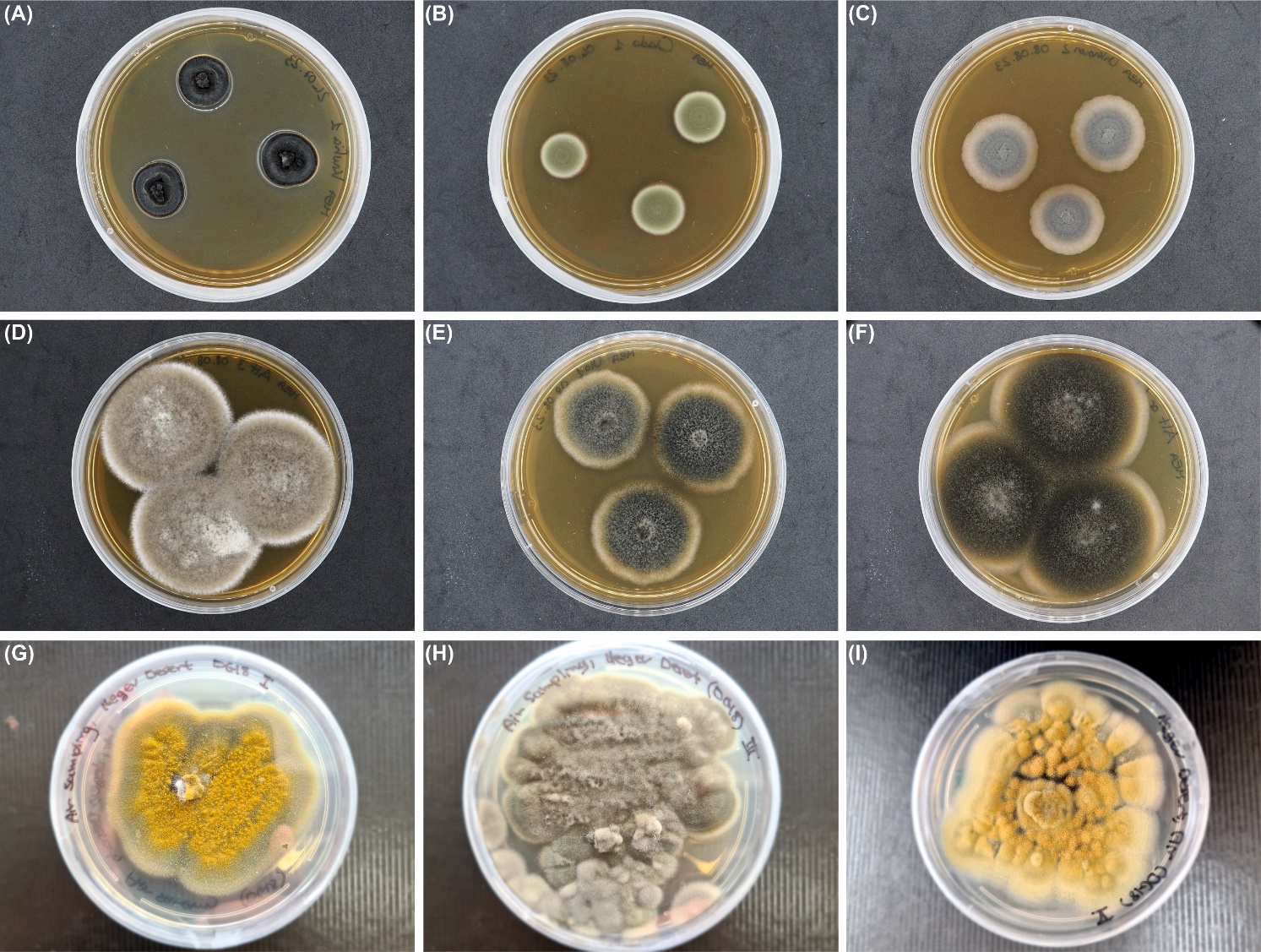


**Supplementary Figure 1**. Pictures of isolated strains from rock, soil and air at the Negev desert petroglyph sites growing on MEA medium (rock and soil A-F) and on DG18 (air G-I). (A) Strain NS14, identified as *Coniosporium* sp., isolated from rock. (B) Strain NS19, identified as *Cladosporium* sp., (C) strain NS17, identified as *Botryotrichum piluliferum*, (D) strain NS10, identified as *Alternaria* sp. in A. sect. Alternaria, (E) strain NS1, identified as *Alternaria* sp. in A. sect. Ulocladioides, (F) strain NS4, identified as *Alternaria* sp. in A. sect. Ulocladium, all isolated from soil. (G) Strain NA1, identified as *C. cladosporioides* complex, (H) strain NA3, identified as *Cladosporium* sp., (I) strain NA5, identified as *Cladosporium limoniforme*, all isolated from air.


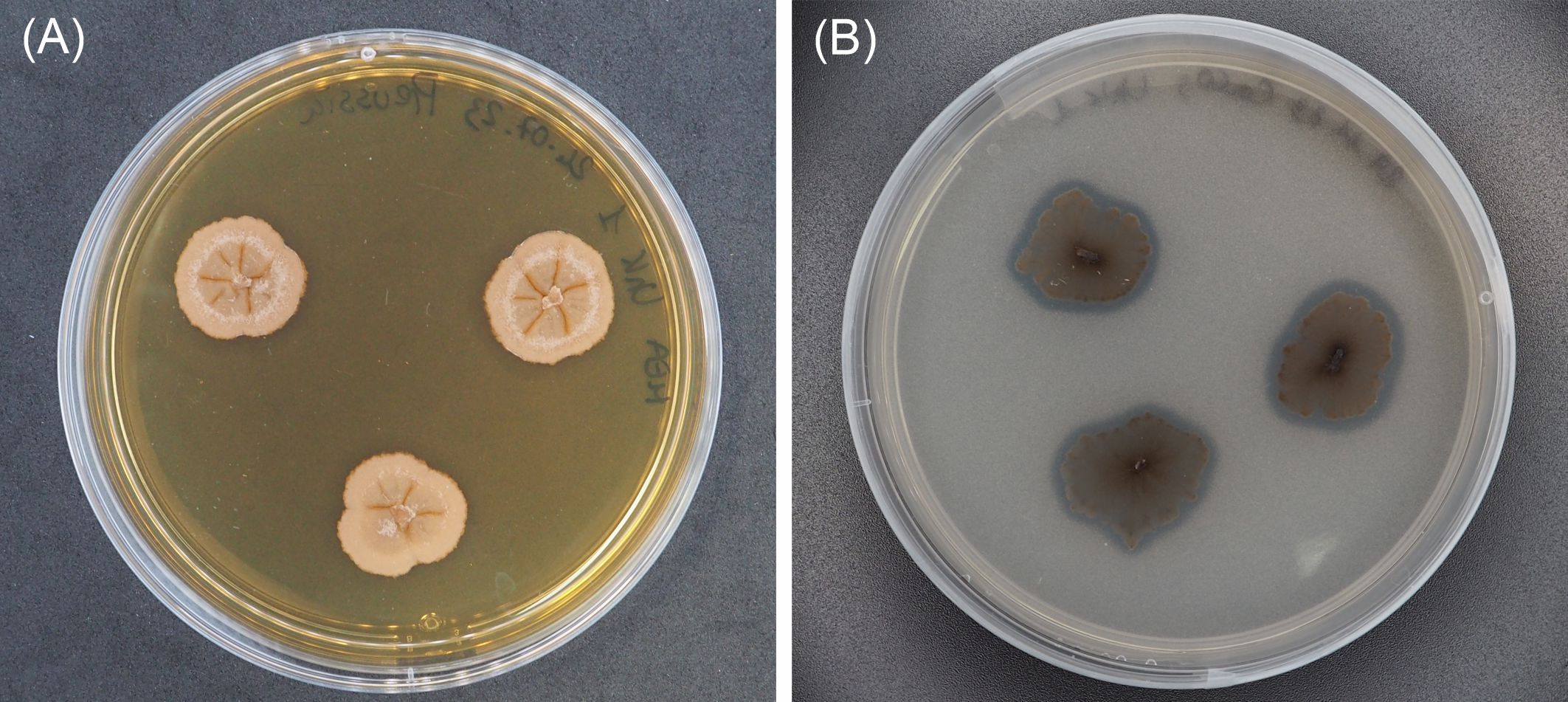


**Supplementary Figure 2**. Strain NS16, identified as *Preussia australis*, isolated from Negev desert soil. (A) Strain NS16 growing on MEA medium, (B) and strain NS16 growing on CaCO3 agar medium showing a clear halo around the colonies (dissolution of CaCO3).


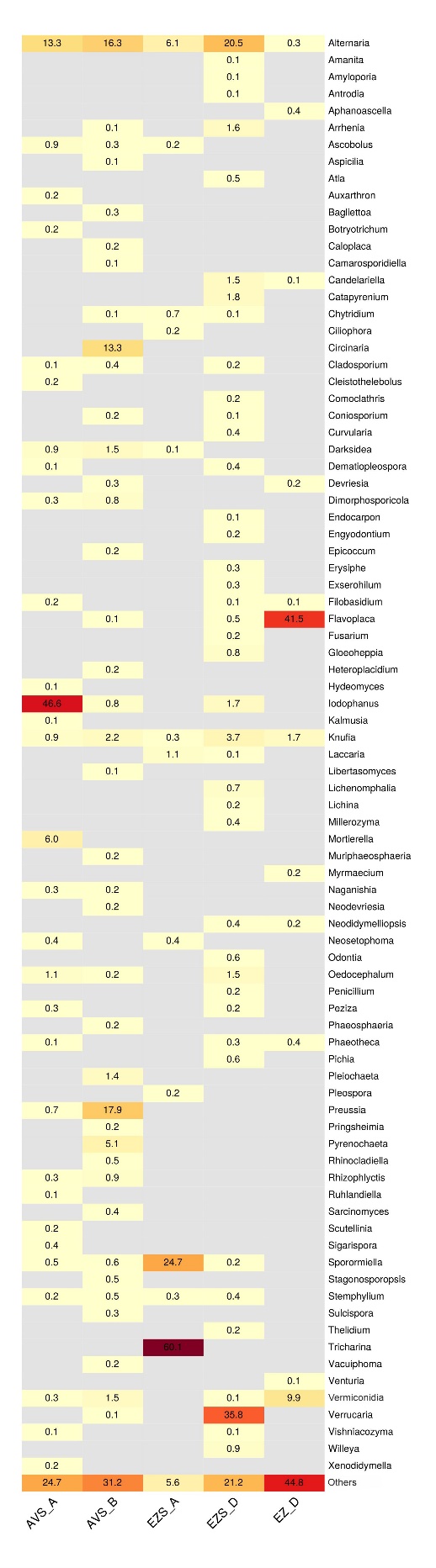


**Supplementary Figure 3**. Heatmap displaying the relative abundance (%) of all classified reads on genus level in each sample (cut-off at 0.1%). "Others" refers to all classifications which have either been detected only under 0.1 % threshold or have not been identified on species level but only on a higher taxonomical level.


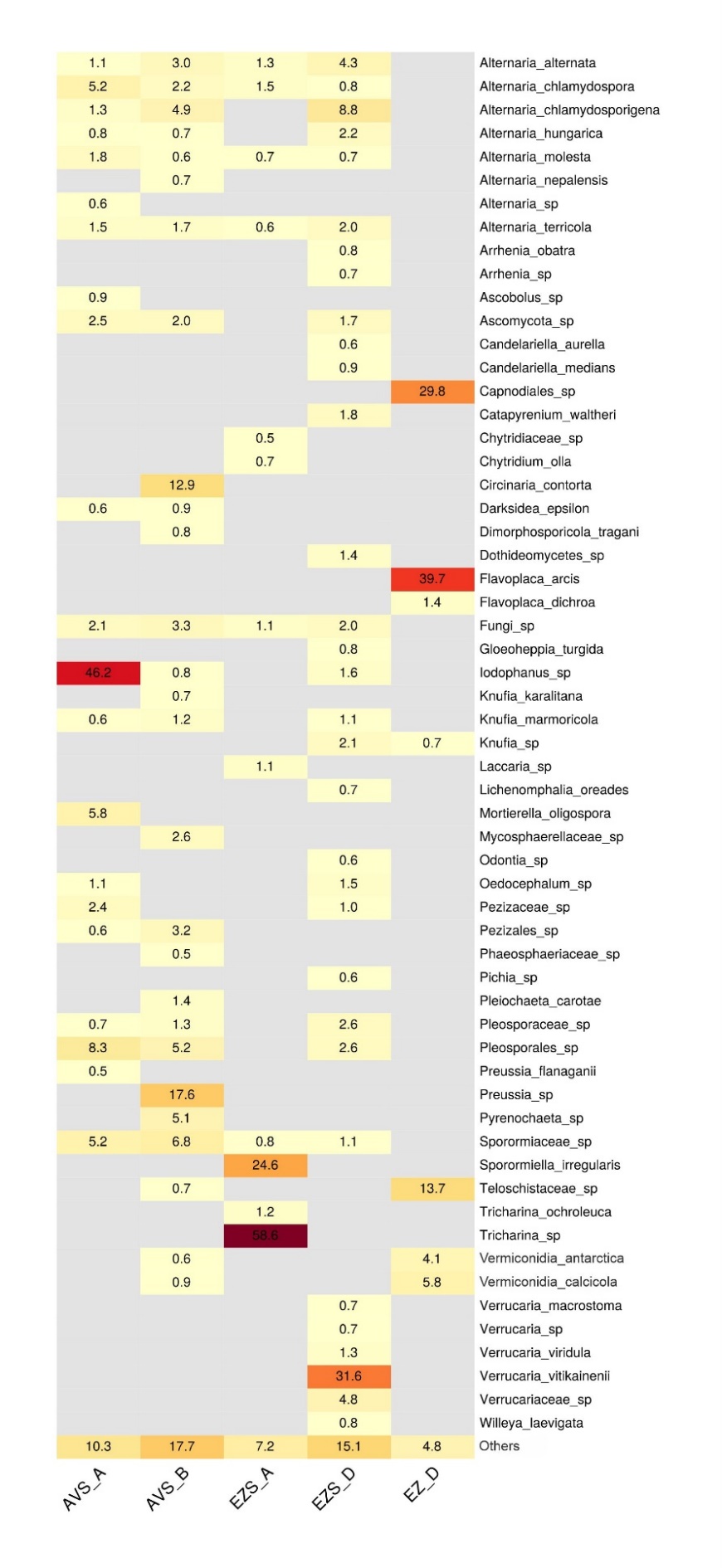


**Supplementary Figure 4**. Heatmap displaying the relative abundance (%) of all classified reads on species level in each sample (cut-off at 0.5%). “Others” refers to all classifications which have either been detected only under 0.5 % threshold or have not been identified on species level but only on a higher taxonomical level.

# Supplementary Tables

**Supplementary Table 1.** List of all isolates from the rock (IR1-IR6) and from the soil (IS61). Isolates IR1-IR5 and IS1-IS49 were divided into 20 groups according to morphology (relabeled NS1-NS20). The number of identical isolates per group is reported together with the total number of isolates per genus. The isolate that was selected to be sequenced per each group is underlined and in bold. At the bottom of the table, isolates that were identified only by microscopy and were not sequenced are also reported.

| **Isolates** | **Groups** | **Number of identical isolates per group** | | **Total number of isolates per genus** |
| --- | --- | --- | --- | --- |
| ***Alternaria*** | | | | |
| IR1 | NS1 | 4 | 32 | |
| IS1 |  |  |  |  |
| **IR2** |  |  |  |  |
| IS2 |  |  |  |  |
| **IS8** | NS2 | 2 |  |  |
| IS9 |  |  |  |  |
| **IS3** | NS3 | 2 |  |  |
| IS4 |  |  |  |  |
| **IS20** | NS4 | 3 |  |  |
| IS21 |  |  |  |  |
| IS22 |  |  |  |  |
| IS6 | NS5 | 2 |  |  |
| **IS7** |  |  |  |  |
| IS10 | NS6 | 3 |  |  |
| IS11 |  |  |  |  |
| **IS12** |  |  |  |  |
| **IS5** | NS7 | 1 |  |  |
| IS13 | NS8 | 5 |  |  |
| IS14 |  |  |  |  |
| **IS15** |  |  |  |  |
| IS16 |  |  |  |  |
| IS17 |  |  |  |  |
| **IS19** | NS9 | 1 |  |  |
| **IS18** | NS10 | 4 |  |  |
| IS23 |  |  |  |  |
| IS24 |  |  |  |  |
| IS25 |  |  |  |  |
| **IS26** | NS11 | 3 |  |  |
| IS27 |  |  |  |  |
| IS28 |  |  |  |  |
| **IS29** | NS12 | 1 |  |  |
| **IS30** | NS13 | 1 |  |  |
| ***Coniosporium*** | | | | |
| **IR3** | NS14 | 2 | 2 | |
| IR4 |  |  |  |  |
| ***Dimorphosporicola*** | | | | |
| **IS31** | NS15 | 1 | 1 | |
| ***Preussia*** | | | | |
| **IS32** | NS16 | 3 | 3 | |
| IS33 |  |  |  |  |
| IS34 |  |  |  |  |
| ***Botryotrichum*** | | | | |
| IS35 | NS17 | 4 | 4 | |
| **IS36** |  |  |  |  |
| IS37 |  |  |  |  |
| IS38 |  |  |  |  |
| ***Xenodidymella*** | | | | |
| **IS39** | NS18 | 1 | 1 | |
| ***Cladosporium*** | | | | |
| IS44 | NS19 | 6 | 11 | |
| IS45 |  |  |  |  |
| **IS46** |  |  |  |  |
| IS47 |  |  |  |  |
| IS48 |  |  |  |  |
| IS49 |  |  |  |  |
| IR5 | NS20 | 5 |  |  |
| IS40 |  |  |  |  |
| IS41 |  |  |  |  |
| **IS42** |  |  |  |  |
| IS43 |  |  |  |  |
| **Not sequenced** | | | | |
| IS50 | *Epicoccum* sp. | | | |
| IS51 | *Epicoccum* sp. | | | |
| IS52 | *Penicillium* sp. | | | |
| IS53 | *Penicillium* sp. | | | |
| IS54 | *Penicillium* sp. | | | |
| IS55 | *Aspergillus* sp. | | | |
| IS56 | *Aspergillus* sp. | | | |
| IS57 | *Aspergillus* sect. *nigri* | | | |
| IS58 | *Aurobasidium pollulans* | | | |
| IR6 | MCF | | | |

**Supplementary Table 2** - Details of the sequencing run performed on the 8 samples (AVS_A, AVS_B, EZS_A, EZS_D from soil and AV_A, AV_B, EZ_A, EZ_D from stone) using the MinIon device (Nanopore sequencing technology).

**Raw reads**

|  |  |  | **Mean** | **Median** | **Mean** | **Median** |
| --- | --- | --- | --- | --- | --- | --- |
| **Sample** | **# of Reads** | **Total Bases** | **Read Length** | | **Read Quality** | |
| AV_A | 407 400 | 82 102 762 | 201,5 | 190,0 | 10,1 | 10,2 |
| AV_B | 532 267 | 108 554 094 | 203,9 | 197,0 | 10,2 | 10,3 |
| AVS_A | 205 400 | 118 379 934 | 576,3 | 681,0 | 11,7 | 11,9 |
| AVS_B | 251 728 | 156 616 223 | 622,2 | 694,0 | 11,7 | 12,0 |
| EZ_A | 23 718 | 4 873 825 | 205,5 | 177,0 | 9,1 | 9,2 |
| EZ_D | 276 262 | 189 628 680 | 686,4 | 731,0 | 11,6 | 11,9 |
| EZS_A | 247 820 | 172 477 346 | 696,0 | 726,0 | 12,0 | 12,3 |
| EZS_D | 232 073 | 153 406 772 | 661,0 | 694,0 | 12,0 | 12,4 |

**Filtered Reads (Q > 9, length 300-900b, adapters/barcodes removed)**

|  |  |  | **Mean** | **Median** | **Mean** | **Median** |
| --- | --- | --- | --- | --- | --- | --- |
| **Sample** | **# of Reads** | **Total Bases** | **Read Length** | | **Read Quality** | |
| **AV_A** | **1 452** | **694 517** | **478,3** | **486,0** | **13,3** | **13,3** |
| **AV_B** | **69** | **28 002** | **405,8** | **362,0** | **12,2** | **11,4** |
| AVS_A | 122 005 | 60 371 155 | 494,8 | 516,0 | 14,2 | 14,2 |
| AVS_B | 176 244 | 83 837 737 | 475,7 | 479,0 | 13,9 | 13,9 |
| **EZ_A** | **115** | **52 325** | **455,0** | **478,0** | **13,4** | **13,6** |
| EZ_D | 210 224 | 108 585 253 | 516,5 | 518,0 | 13,3 | 13,3 |
| EZS_A | 193 898 | 101 834 167 | 525,2 | 496,0 | 14,0 | 14,1 |
| EZS_D | 188 339 | 87 922 283 | 466,8 | 464,0 | 14,2 | 14,3 |

**Reads after Classification:**

|  | **Assigned** | **Unassigned** |
| --- | --- | --- |
| **Sample** | **# of Reads** | |
| AVS_A | 119 791 | 2 214 |
| AVS_B | 154 359 | 21 885 |
| EZ_D | 187 740 | 22 484 |
| EZS_A | 178 546 | 15 352 |
| EZS_D | 29 041 | 159 298 |
